# The river Dniester valley: a long record of late-Cenozoic fluvial evolution within the Eastern Carpathian foreland and East European Platform margin

**DOI:** 10.1098/rsos.250523

**Published:** 2025-10-15

**Authors:** Andrei V. Matoshko, Philip Gibbard

**Affiliations:** ^1^ Independent Researcher, Dniprovoi Chaiky Str. 76, Hermanivka, Obukhiv District, 08753, Ukraine; ^2^ Scott Polar Research Institute, University of Cambridge, Cambridge, UK

**Keywords:** degrading valley, foreland basin, alluvial deposits, river terraces, morphotectonics, rocks erodibility, base-level oscillations

## Abstract

The Dniester valley is a spectacular example of a degrading bedrock fluvial system at the contact between the East European platform and the Carpathian orogen. This study is based upon a combined lithofacies–architecture–morphological study. The complex approach replaces an erstwhile conventional pure geomorphological one, eliminating the shortcomings and inaccuracies, providing a more justified stratigraphy and extended version of the valley evolution. The history of the valley and associated fluvial systems (alluvial fans, delta, coastal alluvial plains) occurred at the end of the Miocene and continued through the Pliocene-Quaternary (11–12 Myr). It unfolded against the background of the retreat of the Eastern Paratethys sea, including the ‘foreland’ and ‘cratonic’ periods and their seven stages. The spatial organization of the river’s drainage networks, sedimentary environments, fluvial styles and landforms changed gradually during these intervals and experienced rapid reorganization when they were replaced. All this left characteristic features within the valley’s four established plain reaches. The tectonic control on these changes through flexural deformation and accelerated uplift/tilt within the platform was decisive while the impact of climatic changes remained problematic. The issues of the river terraces correlation, base-level oscillations, influence of the rock’s erodibility and non-fluvial processes are also considered.

## 1. Introduction

The river valley is one of the most valuable repositories of geological history within the continents [1,2]. It combines evidence of the host’s alluvial deposits within the valley’s morphology which together reflect the interplay of the intrinsic proper river processes with regional and continental scale extrinsic factors such as tectonics, topography, bedrock and palaeogeographical changes, with local subaerial processes in the valley and corresponding river basin. The Dniester valley is well suited in that, unlike the majority of such systems, it preserves the long, 10^7^ year record (from the Late Miocene through the Pliocene to Quaternary times) and evidence of fluvial deposition to the east from the Carpathian Mountains in Europe.

As early as 1869, Barbot-de-Marni [3], together with numerous geologists from Russia, Poland, Romania, Moldova and Ukraine investigated the Dniester valley in search of new discoveries [4–13]. In their investigations, these authors made many assumptions regarding the valley’s evolution, its alluvial deposits and the trend of its development. However, this did not translate into a coherent nor even a generalized picture. The previous research used paradigms formed in the last century, which today are largely outdated. This has resulted in many issues requiring clarification and refinement.

The present study aimed to bridge many of the existing gaps, thoroughly revising the existing database and drawing on its own original data on the architecture of the valley and its alluvial deposits. For this purpose, modern approaches and techniques were applied. By now, two main approaches to studying fluvial archives had been developed, with geological and geomorphological methods prevailing. The first are universal in nature and applicable to rock strata of any age (e.g. [14, with references herein]), while the second are important for fluvial formations expressed in modern relief (e.g. [15, with references herein]). These methods are rarely applied together and to the full extent of their capabilities. This study attempts to show how effectively such a combination can work to reconstruct fluvial history.

The data collected are linked to the substantial volume of the actual published results. All this has led to the first complex synthesis of the Dniester valley evolution. Much of the new evidence concerns alluvial lithofacies and architecture of the valley, as well as new views on the macro-scale dynamics of the fluvial system, its relation to foreland and cratonic tectonics, palaeogeographical and base-level changes. It also includes the influence of bedrock erodibility and secondary processes in the valley that are beyond the scope of this case study and can be of interest to the wide circle of specialists. Paraphrasing the statement [16], the studies of long-time span late-Cenozoic fluvial systems are especially critical because they can provide realistic analogues for the interpretation of the pre-late-Cenozoic rock record.

## 2. General setting

**Figure 1.**
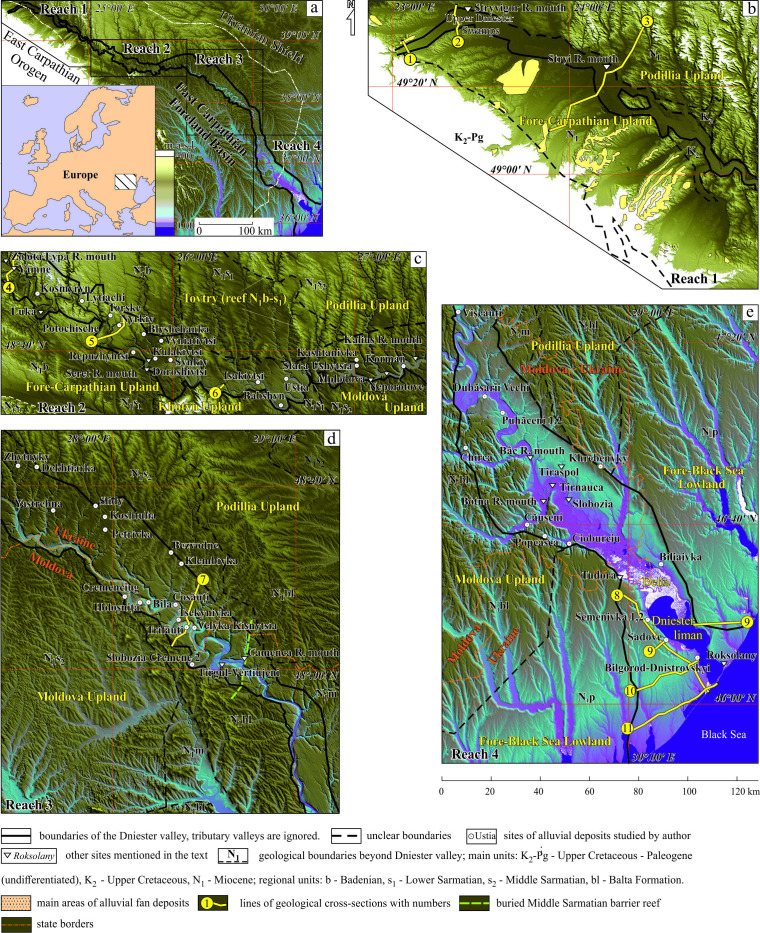
Location map of the Dniester valley and maps of the valley’s reaches. The co-ordinates and type of sites studied by the authors are given in the electronic supplementary material, S1. (a) – general location; (b) – Reach 1, (c) – Reach 2; (d) – Reach 3; (e) – Reach 4.

The Dniester is a medium-sized, continental-scale river in Europe that flows through Ukraine and Moldova into the Black Sea (figure 1). Its modern catchment basin (72 100 km²) occupies part of the Carpathian Mountains, their foothills (Fore-Carpathian Upland) and adjoining uplands (Podillia, Khotyn, Moldova) belonging to the East European Plain. The highest surfaces of the uplands reach 300−500 m above sea level (a.s.l.). The Dniester itself occupies a deeply incised valley with a deep canyon. This paper examines the most informative foothill-plain reaches of the Dniester valley (about 700 km). As far as the mouth of the Stryi River the modern Dniester is a seventh-order stream according to criteria [17], acquiring its eighth-order downstream of this confluence. As much as half of the river’s runoff is derived from the Carpathians and the substantial remaining part lies within the Fore-Carpathian and Podillia uplands.

Regarding the sediment sources, the modern Dniester can be termed a tributary-transfer river system. Within its foothill-plain course, it includes braided, anastomosing (Upper Dniester Swamps), braided-meandering, meandering and anastomosing (near mouth) reaches, within which gravel-, sand- and mud-dominated alluvial deposits occur. The Dniester terminates in a small muddy delta infilling the Dniester Liman (the local name of the estuary).

Based on contemporary ideas (e.g. [18–20]), the largest part of the Dniester fluvial system occurs within the East Carpathian fold-thrust belt and related retro-arc foreland basin. This belt developed from the Late Cretaceous Epoch resulting from the collision of the Tisza–Dacia and Alps–Carpathian–Pannonian microplates against the East-European Craton. The collision conditioned subduction and detachment of the East-European lithospheric slab as well as extension and thrusting in the overriding plates. It ceased in the Late Miocene (11–12 Ma). Beginning in the late Langhian Stage, the collision was accompanied by development of the East Carpathian foreland basin and experienced two phases: asymmetric subsidence [21] and then—differential uplift and exhumation [19,20].

The sedimentary and tectonic processes occurred against the background of the palaeogeographical changes. The most important of these resulted from the recession of the Eastern Paratethys sea [22]. This water body occupied the foreland, its oscillated regression occurred towards the modern Black Sea basin, beginning in the Tortonian Stage [23].

The modern Dniester occurs within the temperate climate conditions, crossing the southern part of the forest zone, forest-steppe zone and steppe. These zones existed in the Pliocene to Quaternary epochs experiencing shifting and transformation, especially with the changes in response to the cold glacial and warm interglacial climatic events [24]. The general cooling trend of the late Cenozoic climate was also associated with a local direct/indirect influence of the Pleistocene continental and mountain glaciations [25,26] as well as with extraglacial phenomena, such as loess sedimentation [27].

The local settings are detailed in descriptions of the valley reaches in the electronic supplementary material, S2.

## 3. Material and methods

This study is based on conventional combined sedimentological, geomorphological and stratigraphical methods with some new elements. The core data were obtained through lithofacies examination of the alluvial deposits. Most were from small outcrops (up to 12 m high and 100 m long) in temporary sand-gravel pits. Natural outcrops are rare since they are rapidly buried by slumped or slope material. The field results were analysed to distinguish the separate lithofacies, facies associations and alluvial architectural elements. All the analyses followed upon advanced sedimentological approaches [14,28–30] applied to the features of alluvial deposits of the Dniester valley with some improved supplements (§§5.1, 5.2 and 5.3).

In principle, the key approach to the study of the fluvial system was by organizing it into reaches, each with its own characteristic structural features and trends of development [31]. After a preliminary analysis of the results obtained, it became clear that the Dniester valley consists of separate reaches (figure 1) that differ in morphology, their alluvial facies assemblages and their high rank architectural elements. The rationale for this division is given in §5.3.1.

The second major part of the data concerns the gross valley architecture. To do this, a topographic study was carried out first by means of construction and analysis of the transverse profiles from 12 to 18 for each of the valley’s reaches. The digital images of SRTM [32] processed by GIS tools and topographic maps of 1 : 100 000 scale were used for relief analysis. The same data and GPS navigation were applied to determine the accurate location of the sites investigated (electronic supplementary material, S1 and table S1.1).

As a result, numerous morphometric parameters of the valley have been obtained, these being included in the overall analysis of the high-rank architecture, together with numerous lithological descriptions of previously studied outcrops and well cores (literature sources and archival, freely available data from the State Geological Information Fund of Ukraine, ‘DVNP Geoinform of Ukraine’). This information was provided for the entire valley during geological surveys. The cores and outcrops with clear location and altitude were checked, selected and reinterpreted in some cases.

The recognition of the high-rank architectural elements [14] was combined with very similar principles and terms (member, formation) of the allostratigraphy in relation to the fluvial geology [14,16,33]. These terms are reasonable substitutes for such divisions including ‘suite’ and ‘series’ which are still in use today for alluvial strata in some countries of the former USSR. This latter terminology was not adopted here since it conflicts with the internationally approved stratigraphic classifications.

## 4. Review

The history of geological studies of the Dniester valley has been presented in many summaries (§1) and records of the Geological Survey (1950s–1990s, archival data, §3). Since part of these records were never published, the current knowledge and the views that were developed in the past are evaluated herein.

### 4.1. Lithological data and provenances

The historical lithological information is abundant and sufficient for the aims of the present study. Gravel, as the typical and predominant component of the alluvial deposits, was recorded by many researchers in all the Dniester reaches. Systematic particle-size analysis, carried out on Holocene alluvial deposits of the Fore-Carpathians, demonstrated their gravel-dominated composition [34]. Local determinations of the gravel clasts’ size, petrographic composition and orientation were made by [35–37] who examined the alluvial deposits of some ancient terraces within Reach 2. In particular, the findings of the crystalline rocks in Reach 2 [37] were associated with so-called ‘mixed gravels’ distributed by meltwaters from the margin of the Middle Pleistocene glaciation [38].

The gravel of the highest terraces of the Dniester was referred [39] to its Carpathian origin, while later [40] two source provenances of the Dniester alluvial deposits were distinguished: the Fore-Carpathians and the so-called ‘platform’, probably meaning the East European Platform. Today, the new evidence available indicates that it is reliable to state about three main source areas. The Carpathian orogen (Carpathian Provenance) is the primary and most important provenance, while the conglomerates of the alluvial fans [41] are an intermediate but direct source for alluvial deposits of the valley’s Reach 1. The content of gravel and sand fractions is defined by the more durable types of the sedimentary and metamorphic rocks, which, in order of their frequency, comprise sandstones (substantially predominant), marls, slates, menilites, quartzite, gneiss and quartz [5,35–37,42]. Most of these lithologies have been derived from the outer orogenic area of the mountain range where they persisted under intense and long-distance transportation encountered throughout the river’s course as far as its mouth.

Down valley, the second most important clastic source is Miocene rocks of the valley proper together with its Podillia tributaries (Podillia Provenance). This includes the grainstones, boundstones, algae balls and crystalline limestone clasts as well as clay balls [43]. Although these rocks are the main substrate underlying this part of the valley, most of them are weak, friable and rapidly fragmented during transport. Meanwhile, there is a more resistant variety initially called the ‘Carpathian Pebbles’, the primary source of which is unknown, although it is also associated with the Podillia Provenance [44]. The granules and pebbles with rare fine cobbles derived from sandstones, siltstones, chert and quartz, compose this assemblage. They are encountered most frequently in the uppermost alluvial units of Reaches 3 and 4, but they also occur with different but lesser content in the lower units. Hereafter, the ‘Carpathian Pebbles’ lithology is distinguished from the ‘true’ gravel assemblage of the Carpathian rocks.

The third provenance is the Meso-Palaeozoic and Pre-Cambrian rocks into which the Dniester valley is incised (Dniester Provenance) in the downstream part of Reach 1 and Reaches 2 and 3. According to [42], these rocks gradually dilute the gravel composition of the Carpathian and Podillia origin at the beginning of Reach 2, their content increasing downstream in Reaches 2 and 3. Here, the most frequent lithologies are flints and opokas of the Upper Cretaceous series, reddish Devonian sandstones, Silurian dolomites and marls and notably Neoproterozoic siltstones [42]. The presence of granites was noted [45] in the basal horizon of the lower conglomeratic unit in Tiraspol. These clasts were eroded from the Reach 3 assemblage, where basement igneous rocks outcrop. After leaving the canyon (Reach 4), the proportion of Carpathian rocks and fewer ‘Carpathian pebbles’ together with Miocene carbonate rocks in alluvial gravel increases again, although here in the finer fractions [46]. The authors’ observations indicate that the clay balls, clay detritus, ‘Carpathian Pebbles’ and rare sandstones of the Balta Formation are characteristic of gravel constituents.

The provenances noted are reflected in the mineralogical composition of the sands ([42,44–46, references therein] archival data). Here, the predominant mineral is quartz, while feldspars (3−11%) appear only in Reach 4 (influenced by the igneous rocks from Reach 3). Garnet is a permanent component of the allogenic minerals in the heavy fraction and occurs in three of the main assemblages (50% or more in content). In Reach 1 (lowest units) it is combined with other minerals (in descending order of abundance), zircon, titanomagnetite, leucoxene, rutile, tourmaline and hornblende, which obviously matches the Carpathian Provenance.

The garnet–ilmenite–leucoxene assemblage (up to 75% of the content) is characteristic of the upper-canyon units of Reach 2 and Reach 3, as well as for the lower units of Reach 4 (Podillia Provenance). This assemblage definitely corresponds to that of the Balta Formation [44]. There are also several assemblages within the canyon units, where the garnet–ilmenite–leucoxene assemblage is diluted by some other minerals derived from the local rocks, including zircon, rutile, disthene, sillimanite, staurolite and allogenic glauconite (Dniester Provenance). Within Reach 4, the content of garnet again increases to 33−56% accompanied by the notable percentage of ilmenite, zircon and leucoxene [40]. According to [42], the following average percentage of the main component minerals of the heavy fraction of the modern Dniester unit, for Reaches 1, 2 and 3: garnet (26.5, 23.4, 17.9), zircon (15.4, 10.9, 14.1), titanomagnetite (14.4, 10.1, 9.5) and leucoxene (13.4, 9.7, 10.3) occur correspondingly, these proportions pointing to dilution of the primary Carpathian assemblage.

Finally, the very minor content or lack of silt-pelite fractions in the sand and gravel deposits at Tiraspol and its surroundings was noted [45]. The mud constituent of the floodplain and abandoned channel deposits from the same sites are represented by hydromica and montmorillonite, where they are also poor in plant matter residues.

### 4.2. Sedimentary processes

All previous researchers unambiguously accepted the alluvial origin of the Dniester sand and gravel deposits. The division of the individual units into groups referred to channel and floodplain environments is also seen as characteristic in many publications and has been adopted by survey geologists.

In previous studies, the existing multiple descriptions of deposits are simplistic and look very similar. The vast majority of outcrops have been studied only vertically in a narrow strip (e.g. [35,36]) in order to determine the sequence of units, i.e. the facies, geometry of alluvial bodies and their architectural elements substituted by definition as a ‘terrace’. This led to the fact that, until recently, the alluvial deposits of the Dniester valley were identified and interpreted from only one sedimentary environment—fragmentary fluvial accumulation under conditions of the transfer river, ignoring possible alternatives.

The lithofacies-architectural study of recent years substantiated the existence of a deltaic environment in Reach 3 [44] and revealed the alluvial fans in Reach 1 [41], which are regarded by some authors [38,47] as ‘normal’ terraces. The erroneous palaeogeographical conclusions that a simplified geomorphological approach can arise from this approach are shown by examples [48] where, in the section of ‘one terrace’, i.e. deposits of those ordinary terraces and alluvial fans alternate.

### 4.3. Stratigraphy

**Figure 2.**
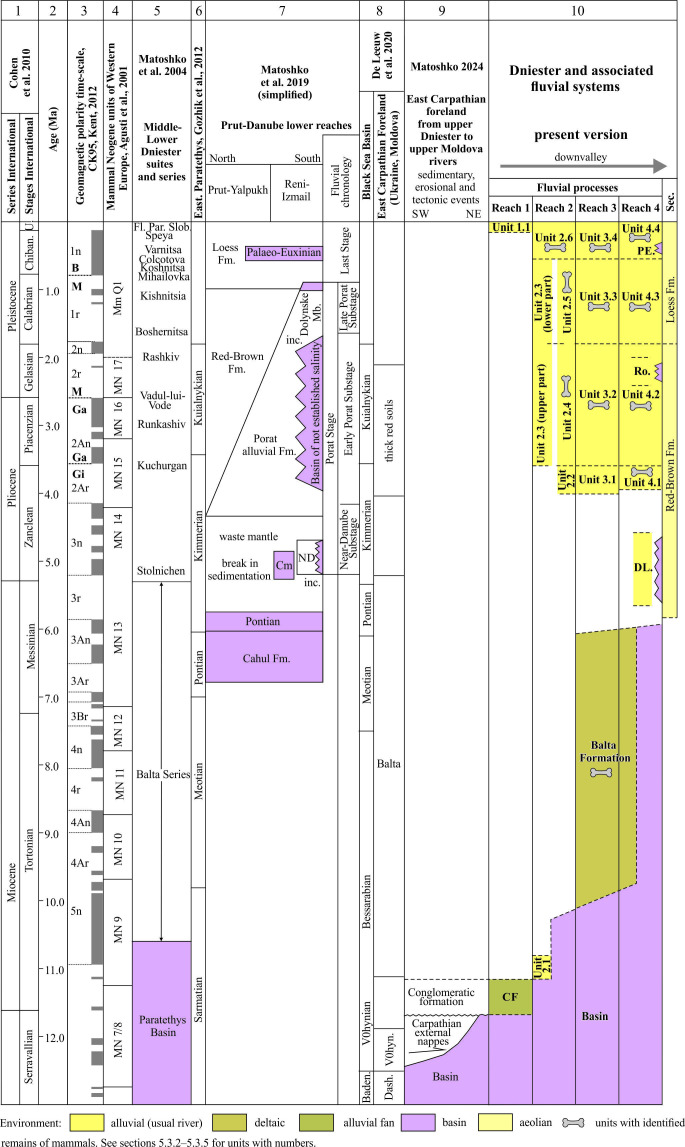
Correlation scheme of the Dniester valley and associated alluvial formations against the background of the tectonic, sedimentary and erosional evolution with regional and general stratigraphical schemes. Abbreviations: Chiban., Chibanian; U., Upper Pleistocene; Fl., Foodplain; Par., Parcani; Sl., Slobozia; O., Odessian; inc., incision; Cm., Cimmerian; ND., Near-Danube; Port., Portopherian; Boshpor., Bosphorian; Baden., Badenian; Volhyn., Volhynian; Dash., Dashava; CF, Congomeratic Formation; Dl., Dniester Liman Series; Ro., Roksolany Suite; PE., Palaeo-Euxinian; Sec., secondary processes. The contents of the columns are given in accordance with 1 and 2 – [50], 3 – [51], 4 – [52], 5 – [12], 6 – [53], 7 – [54], 8 – [23], 9 – [41].

It appears that the Dniester terrace staircase is the longest and most complete terrestrial stratigraphical record of the late Cenozoic in the East European Platform (e.g. [12,13,49]). The analysis of the available stratigraphical evidence shows that most of the terrace accumulations are associated with Reach 4, less so with Reach 3 and a small part with units of Reach 2, i.e. the valley is quite variably studied in this regard. The stratigraphical record of the Dniester valley has relied upon terrace age-elevation data, index beds, biostratigraphical evidence, thermoluminescence and ^14^C numerical dating, together with magnetostratigraphical interpretations (figure 2). There are also numerous value judgments concerning the age of certain alluvial deposits or terraces, which are only of historical interest.

The recognition and correlation of river terraces was previously the dominant morphostratigraphical method employed within the Dniester valley. It was based on the relative altitudinal position of the terrace (its sole) above the mean water level in the river. There were significant shortcomings to this seemingly conventional approach. The determination of the altitude concerning exposures was made, at best, by means of the medium-scale topographic maps by sight, while the boreholes were tied geodetically. Most of the published valley cross-sections are schematic or composite and are based only on available borehole data without correlation to outcrops [12,55]. However, the identification of terraces was supported only by visual observation [56]. The debate over rival terrace schemes and the finite number of terraces (from 6 to 14 in the altitudinal range of up to 250 m) remains unresolved. Lately, the ‘numerical’ approach was revived [38,47] without the addition of new supporting arguments. The unified principles for mapping terraces have remained unstandardized. All this has led to confusion.

At the same time, some detailed studies [8,57] have revealed the presence of adjacent terraces, the bases of which differ by 1−2 m and the units separated by clear lateral unconformities. Many examples include that the number of terraces increased with decreasing distance between boreholes or outcrops and raises the question of their final number.

The correlation of terraces determined in one place or reach was often extended to other reaches (e.g. [5,6,42,52]) *a priori* implying a cyclic nature of terraces throughout the whole valley. Moreover, the numerical approach has been extended for intervalley correlation belonging to different river basins [9,11,38,58]. The validity of such correlations is highly questionable. Most noted problems are characteristic for analogue studies worldwide and they do not yet have a universal solution [59].

The most general age limits are set by marker beds underlain or overlain by the alluvial units of the Dniester valley. The most reliable are regional basin strata of the Late Miocene (Sarmatian and Lower Pontian regional stages, figure 2) based on calcareous nannofossils, foraminifera and ostracods [60] and the alluvial-deltaic Balta Formation of the Middle Sarmatian–Meotian–Lower Pontian regional stages based on mammal remains [23,44]. The Lower Sarmatian age of the alluvial Conglomeratic Formation rests on the solitary fact of separation of two units of conglomerates by the basin clays that yield a foraminiferal association, including *Elphidium rugosum* [61]. The more ancient part of the alluvial units of the Dniester River basin is overlain by the deposits of the Red-Brown Formation of unidentified origin. Their base in the adjacent basin of the Prut River is dated approximately to the upper part of the Zanclean Stage [54]. The top of the reddish-brown palaeosols at the base of the loess sequence at Roksolany lies slightly lower than the Brunhes/Matuyama geomagnetic chron boundary [62,63], with an age of approximately 1 Ma (late Early Pleistocene Subseries).

The Dniester alluvial deposits contain various and numerous types of fossils, the finds of most of which have palaeoecological and palaeogeographical implications. Among them, only remnants of mammals derived from the alluvial deposits [6,8,10,40,64–68] have biostratigraphical significance. All these data were previously summarized [12,13,49]. The number of qualified finds is not as great as it might seem from the primary long lists of the fauna recovered. A part of mammal species is lacking in the international palaeontological databases and is not bound to much more universal and spatially wider biozones.

Alluvial deposits from the several sites within Reaches 3 and 4 have been thermoluminescence dated [10,69]. A total of 15 age determinations were obtained, from these 13 (whose locations are identified) in the interval 1.1–0.2 Ma. The dating of fluvial deposits is lacking in the modern overviews of possible applications of the thermoluminescence methods, and most of the ages obtained are beyond the limits of the method [70]. Therefore, these results should be considered questionable.

Previously, palaeomagnetical studies were also carried out for some alluvial units [10,71,72]. They recorded separate episodes of magnetization change within short (10^−1^ m) alluvial sections, the significance of which do not have independent stratigraphical importance.

Several ^14^C dates have been obtained for silty hillslope (loess) deposits overlying alluvial deposits of the uppermost level of the so-called second terrace) of Reach 2 in the Molodova and Korman Late Palaeolithic sites [8,57]. They indicate the Late Pleistocene age of this level (>45 ka). This was later confirmed for Molodova by Haesaerts *et al.* [73]. The same authors gave ^14^C dates (>20 ka) for the most ancient loess blanket overlying alluvial deposits of the so-called first terrace in the Cosăuţi and more than 23 ka in the Doroshivtsi [74]. These results have been integrated in the current study.

### 4.4. Ideas concerning the valley’s development

The first complete concept of the evolution of the Dniester was outlined by Vyrzhykovskiy [4], based on the study of the left bank area within Reach 3. His history began from the Balta Stage onwards, i.e. since the Pontian. The deposits of this stage were considered to be a product of the huge delta fed by precursors of the Dniester and Prut rivers, which predated the rivers’ more ancient history. Further evolution consisted of directed more or less uniform entrenchment and advance of the valley in a south-southeast direction under the conditions of the basins’ retreat and oscillations during the Pliocene Epoch and Quaternary Period. These simple ideas were shared by most of the researchers [12,13].

They were essentially added by recent study results on the Conglomeratic Formation [41], Balta Formation [44] and in the concept of the evolution of the fluvial system of the East Carpathian foreland [23]. In particular, the studies of these formations have extended the history of the Dniester basin area back over 5 Myr (from the Lower Pontian to the Lower Sarmatian) that is developed further here.

Thus summarizing the review presented, the historical lithological information is sufficient for aims of the present study while other considered aspects require revision, correction and supplement.

## 5. Results

### 5.1. Lithofacies

The lithofacies study has been applied to deposits whose alluvial origin was unequivocally recognized in previous research. This refers to a wide spectrum of clastic rocks, from mud to boulder classes. Their division is based on grain-size and sedimentary structures (table 1) with the additional characteristics of the pebble fabric for the gravels.

**Table 1.** Facies of alluvial deposits (codes are according to [14]).

facies, codes	dominated grain-size range	sedimentary structures	thickness m	name of sites according to figure 1 where facies are studied	interpretation (modes of transport and deposition)
conglomerate massive clast- and matrix-supported Gm^a^	– 4 to −6 φ	absent; inverse grading, common orientation of elongated clasts and faint cross-bedding in places	0.05−0.4	Torske, Nyrkiv, Blyshchanka Vyniativtsi, Kulakivtsi, Synkiv, Isakivtsi, Babshyn 2, Tsekinivka, Slobozia Cremene 2, Puhăceni 2	hyperconcentrated flow; traction bedload
conglomerate massive matrix-supported Gmm	– 1 to −5 φ	absent	>1.2	Kosmyryn, Nyrkiv Potochische, Velyka Kisnytsia, Vîscăuți, Cioburciu, Semenivka	plastic debris flow
conglomerate massive clast-supported Gcm	– 1 to −5 φ	absent	0.1−0.4	Ustia, Vîscăuți, Salcia, Cioburciu	water-current flow flow; bedload
conglomerate stratified clast-supported Gh	– 3 to −8 φ	faint horizontal or inclined parallel bedding	0.2−16.0	Lytiachi, Repuzhyntsi, Isakivtsi, Babshyn 1, 3, Kashtanivka, Neporotove, Cosăuţi 2, Bezvodne, Trifăuți, Slobozia Cremene 2	hyperconcentrated flow; traction bedload
conglomerate stratified clast-supported Gt	– 3 to −7 φ	trough cross-bedding	0.2−2.0	Lytiachi, Neporotove, Puhăceni 1, Slobozia Cremene 2	water-current flow; bedload
conglomerate stratified clast-supported Gp	– 4 to −7 φ	planar cross-bedding	2.0−2.5	Neporotovo, Trifăuți, Holoșnița	water-current flow; bedload
sand stratified St	3 to 0 φ	trough cross-bedding	2.0−6.0	Lytiachi, Babshyn 2, Stara Ushitsia 2, Zhytnyky, Dekhtiarka, Yastrubna, Bezvodne, Klembivka, Slobozia Cremene 2, Dubăsarii Vechi Puhăceni 1, Chirca, Khrebenyky, Salcia, Căușeni, Popeasca, Biliaivka	water-current flow; bedload
sand stratified Sp	3 to 1 φ	planar cross-bedding	0.5−5.0	Lytiachi, Ustia, Slidy Yastrubna, Holoșnița, Cremenciug, Cioburciu, Biliaivka	water-current flow; bedload
sand stratified Sh	1 to –1φ	horizontal bedding	1.0−2.0	Vykhvatnivtsi, Neporotove, Yastrubna, Cioburciu, Biliaivka	water-current flow; traction bedload
sand stratified Sl	4 to 2 φ	low-angle cross-lamination (bedding)	0.1−0.5	Slidy, Petrivka, Cremenciug, Puhăceni 2, Cioburciu, Biliaivka	water-current flow; bedload
sand stratified Sr	4 to 2 φ	ripple cross-lamination	0.2	Lytiachi, Chirca	water-current flow; bedload
sand massive and stratified Sm	4 to 2 φ	absent; faint planar lamination	0.4−2.0	Torske, Nyrkiv, Blyshchanka, Vyniativtsi, Kulakivtsi, Synkiv, Isakivtsi, Babshyn 2,3, Slobozia Cremene 2, Puhăceni 2	hyperconcentrated flow, water-current flow, still water; suspension load
mud stratified Fl	>4 φ	faint parallel stratification	0.05−2.0	Trifăuți, Cremenciug, Vîscăuți, Dubăsarii Vechi, Chirca, Salcia	still water; suspension load
mud massive Fsm		absent		Babshyn, Neporotove, Zhytnyky, Yastrubna, Koshtulia, Dubăsarii Vechi, Chirca, Cioburciu, Biliaivka	

^a^ Introduced by author.

**Figure 3.**
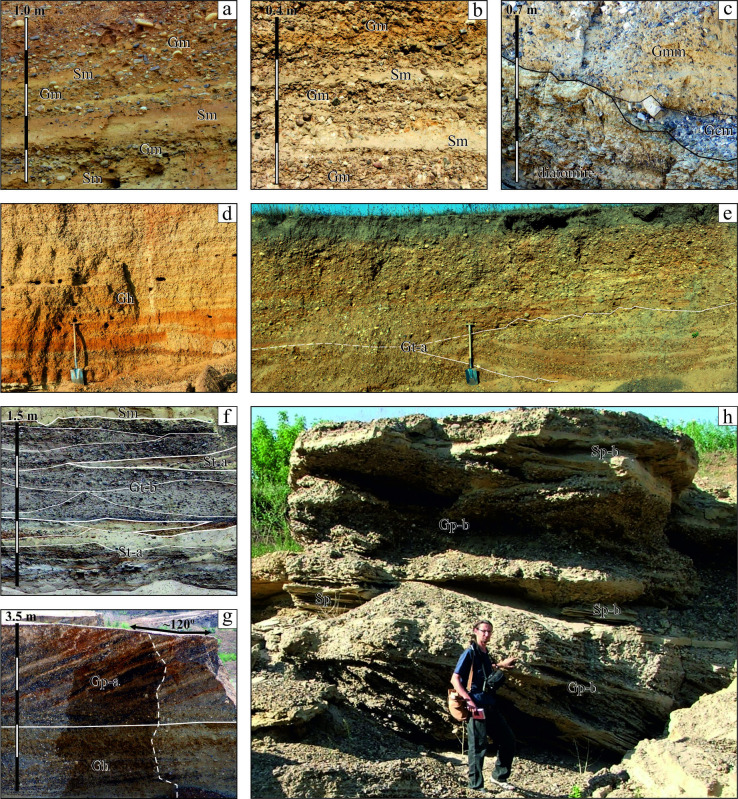
Gravel-dominated facies: (a,b) massive matrix- to clast-supported conglomerate with lenses of massive or faintly laminated sand ((a) Kulakivtsi and (b) Tsekinivka)); (c) massive matrix-supported conglomerate overlain by lag clast-supported conglomerate and bedrock, Vîscăuți; (d) horizontally bedded clast-supported conglomerate, Kashtanivka; (e) large-scale clast-supported trough cross-bedded conglomerate with sand lenses parallel to trough sole, Lytiachi; (f) small-scale matrix-supported trough cross-bedded conglomerate alternating with trough cross-bedded sand, Slobozia Cremene 2; (g,h)– clast-supported planar cross-bedded conglomerate with unidirectional bedding dip ((g) solitary set with straight regular beds, Trifăuți; (h) tabular irregular conglomeratic sets cutting each other and interbedded with planar cross-bedded sands, Holoșnița). The length of shovel is 1.1 m, the height of man is 1.78 m.

**Figure 4.**
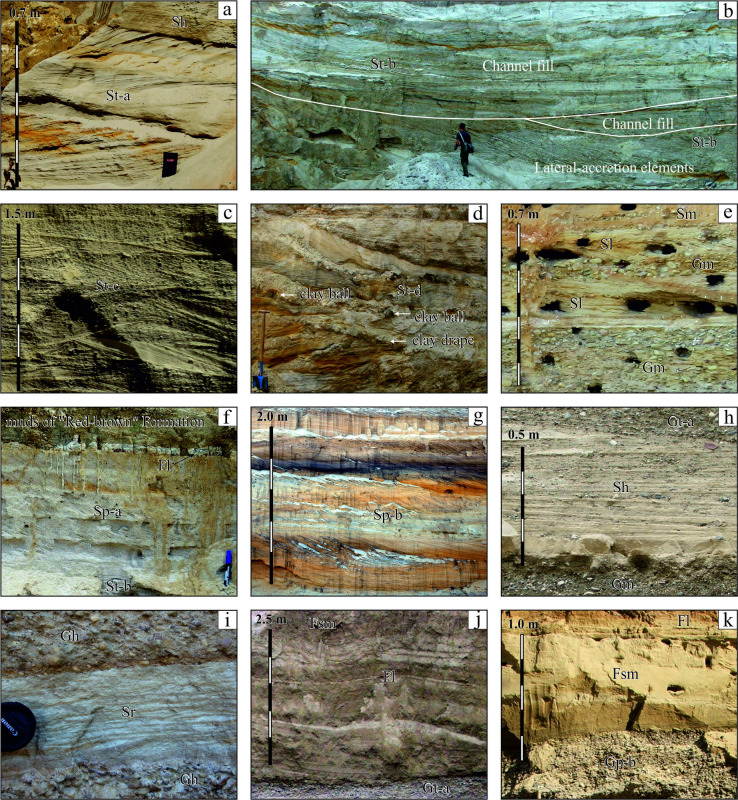
Sand-mud dominated facies: (a,b,c,d) trough cross-bedded sands ((a) well sorted sands in meter-scale long sets, Klembivka; (b) variously sorted sands in tens of metres long regular sets, Căușeni; (c) moderately-poorly sorted gravelly sands in regular short sets, Slobozia-Cremene 2; (d) poorly sorted clayey sands with mud drapes in irregular sets with curved beds and bounds, Chirca); (e) low-angle (<15˚) cross-bedded sands in lenses amid conglomerates, Synkiv; (f,g) planar cross-bedded sands with unidirectional bedding ((f) regular thin sets with straight large-slope beds, Yastrebna; (g) irregular sets with different shapes (straight and sigmoidal) and slopes of beds, Cioburciu); (h) horizontally bedded poorly sorted sands in solitary member amid conglomerates, Neporotove; (i) lens of ripple cross-laminated fine and very fine sand in conglomerates, Lytiachi; (j) wavy interbedding of sand and mud, taking uppermost position in the section of alluvial member, draws attention unusual angular wedging out of beds at the lower base with conglomerate, Vîscăuți; (k) thinly horizontally bedded mud overlying massive mud and that in turn by conglomerate, Holoșnița. The height of the man is 1.8 m, the length of shovel is 1.1 m, the length of marker is 14 cm, the diameter of lens cap is 5 cm.

Hereafter, the modified Udden-Wentworth’s grain-size classification [75] is used. The non-sedimentary structures (water-escape plications, low-amplitude faults, clast coatings and saturation of the matrix with oxides of iron and manganese) are also distinguished. With one exception, the names of the facies and references to their codes follow Miall [14]. The facies are discussed in three groups where the dominant components are gravels, sands and muds. The examples of facies are shown in figures 3 and 4 and detailed descriptions of most gravel and sand facies are cited in the electronic supplementary material, S3.

The first group consists of a massive clast- and matrix-supported (Gm), massive matrix-supported (Gmm), massive clast-supported (Gcm) and stratified clast-supported (Gh, Gt, Gp) gravel-dominated facies. The induration of coarse deposits varies from gravel to conglomerate. The degree of packing and lithification in many cases changes over short distances. Proper gravel can often be seen in the fresh exposures of an operating pit and only conglomerate in some abandoned exposures. Some units are represented only by conglomerates. In the grain-size scale, the gravel facies occur mostly in range from granule to very coarse cobble. There are also rare finds of fine boulders. The flat limestone clasts (1–4 cm thick) reach long axes of 50 cm. The content of gravel varies from 30% (conditional content which differs gravel from gravelly sand [29]) up to 90% (source: archival data).

The roundness of clasts often varies depending on the ratio between hard, far-transported Carpathian and local rocks on the one hand and friable local rocks on the other (§4 above). For harder rocks, a discoid and oblate shape is predominant, followed by flattened, prolate, the blade-like shape is characteristic for the less durable clasts. Locally, where the angular and subangular clasts make up more over half of the pebble content, the coarse sediment can be referred to as a breccia. The gravel facies are divided into massive and stratified varieties and those, in turn to matrix- and clast-supported units (figure 3). In case of the Dniester gravels, the matrix of the particles is generally more than 2 mm, i.e. sand and mud.

The sand-dominated (stratified trough cross-bedded (St), low-angle cross-laminated or thin cross-bedded (Sl), massive or faintly planar laminated (Sm), ripple cross-laminated (Sr), planar cross-bedded (Sp) and horizontally laminated or thin bedded (Sh) facies are represented in figure 4a–h. They are similar by their features and compared to analogues from other sandy rivers of the East-European Plain, with comparable order and discharge [12]. However, they differ by the notable common coarseness and almost ubiquitous inclusions of gravel. Their vast majority are stratified sands while massive examples rarely occur. The interpretation of sand facies is much clearer than gravels and more definitely reflects the modern concepts of transport/deposition.

There are two mud-dominated facies (>4 φ) with relatively low occurrence: Fl (planar parallel thin bedded or laminated, figure 4f,j,k), alternated with sand in places and Fsm (massive), the only ones in which gravel is almost never found. They form thin (first tens of centimetres thick) sheets (figure 4k) or drapes (figure 4d,f), some of which are more or less modified by pedogenesis.

Both facies originated under conditions of waning flow or still water as a result of settling from suspension.

### 5.2. Lithofacies associations

**Figure 5.**
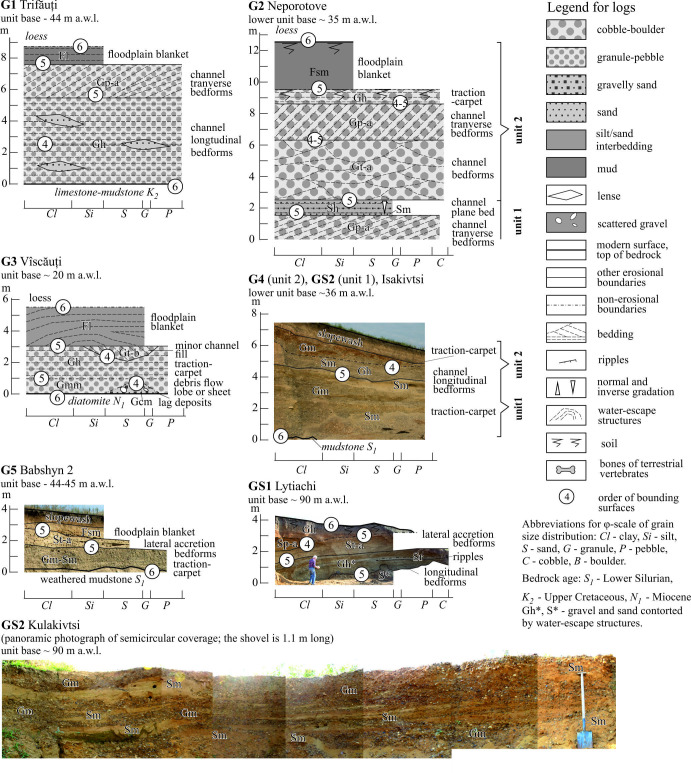
Log-examples of the gravel-dominated and gravel-sand-dominated associations. See location in figure 1.

**Figure 6.**
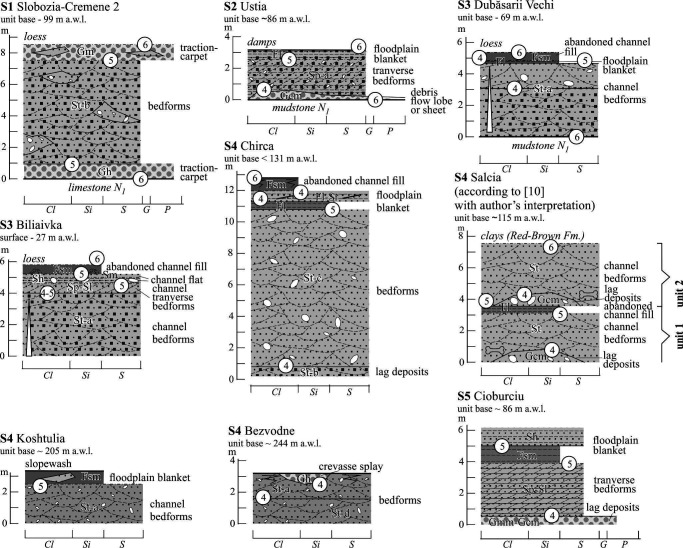
Log-examples of the sand-dominated associations. See legend in figure 5 and location in figure 1.

For alluvial facies, their repeated combinations are typical. In the Dniester valley, there is a great diversity of facies, which are divided into gravel-dominated (G), gravel-sand (GS, gravel/sand ratio is changeable in lateral and (or) vertical direction) and sand-dominated (S) associations which occur. These facies associations reflect a significant difference in the conditions of sedimentation and, above all, the flow competence. Their general characteristics are presented here. Their detailed description and interpretation are cited in figures 5, and 6 and the electronic supplementary material, S4.

The thicknesses of associations vary from 2 to 12 m. Most contacts between facies in the gravel-dominated and sand-gravel associations are clearly erosional, sub-horizontal (G1–G3, G5) and inclined (G4, G6, GS1). The GS2 association has lenticular arrangement. The facies contacts in the sand-dominated associations are erosional; sub-horizontal, inclined and concave in case of channel environment, while often non-erosional and predominantly horizontal in case of floodplain and abandoned channel environment.

Among the environments noted the channel predominates. Some associations (G4, GS2, S1, part of electronic supplementary material, S4) represent only this. This is usually associated with non-periodic annual floods of different duration and intensity. The channel group often begins at the base, with a coarser and/or less sorted facies, including typical ‘lag’ deposits. Further upwards stacking of facies types in the gravel-dominated successions does not reveal patterns. In some sand-dominated associations, a clear trend of decreasing sand grain size towards the top has been recorded. There too the Sp facies often follows St facies or complete channel sections. This is associated with differences in duration changes in flow regime [14]. It is likely that part of traction carpet facies and debris-flow deposits represent non-channelized ‘sheet’ streams like those within alluvial fans [41].

The floodplain group of facies are subordinate in nature, however, in this case, they are differentiated within vertical sections, representing more or less stable seasonal flood deposition. It is noteworthy that facies of abandoned channels are rare and thin. This, as well as the absence of palaeosols in floodplain facies, attests to the absence of free meandering and stable position of channel or channel belt.

Part of the associations can be termed ‘elementary’ or those reflecting single flood events, while others are composite, with a vertical accretion trend. The accretion may concern only stacked channel facies (most widespread case, G1, G2, G3, S1, S4), stacked floodplain facies (S3) or combined cycles (S2, S4). Such accretionary complexes could accumulate over several events during much longer periods.

### 5.3. Architectural elements of alluvial deposits

#### 5.3.1. General characteristics

The architecture of alluvial deposits is part of the general architecture of the valley. Their elements are sedimentary bodies (lithosomes) characterized by shape, orientation, areal extent and position in the definite hierarchy. The latter is determined by recognizing the surfaces that bound the elements and assigning them a rank in the form of an ordinal number from smaller to larger. As noted above, the present study authors adopted Miall’s classification of architectural elements associating alluvial bodies at a high rank with informal allostratigraphic divisions [14].

The present approach is less detailed, determined mainly because of the limited size of outcrops and those with differently orientated walls for three-dimensional visualization. However, it allows combining the architectural elements with separate facies, their associations and main environmental groups of facies. In this sense, the interest lies in the architectural elements of medium-high rank beginning from the surfaces of the fourth order. The rank and characteristics of the same surface’s orders (figures 5 and 6) is somewhat different from Miall’s [14, table 4.2].

An architectural analysis involves reconstruction of the direction of the palaeocurrents. In this case, the authors used the dip directions of cross-bedding in Gp and Sp facies. The number of suitable sites for these measurements is rather restricted and single values obtained give arbitrary results. Moreover, this randomness is naturally related to the variable directions within meanders. For the purposes of the present study, it turned out to be much more reliable to focus on orientation of the valley, stretch of the terraces and incised meanders.

The medium rank architectural elements of the fourth order confine predominantly meso-bedforms and those of fifth order—macro-bedforms and groups of the environmental facies. They have intermediate value for our analysis and are considered in the electronic supplementary material, S5.

The sixth-order surfaces represent the base-top of alluvial cycle or cycles, i.e. boundaries of alluvial allostratigraphic members (§3), referred also to the separate river terraces or one accretionary unit. These boundaries are distinguished not only by the results from outcrop studies, but also by interpretation of more numerous and long borehole sections. They are mostly horizontal or gently inclined towards the shifting valley axis.

The thickness of members varies along the vertical profile of the valley and between its lateral extents from 1−2 to 12 m (one cycle) and up to 23 m (stacked cycles), respectively. There is primary depositional thickness of members (that can be distinguished only in exposures) and secondary depended on truncation by erosion. Wherein the primary thickness can be related to change in the flood discharge, stream competence and value of the transported load, as well as to the trend of accretion and superimposition of cycles.

The extent of the preserved bodies is also quite different (10^1^–10^4^ m) scroll-like in plan segments within incised meanders to long (10^3^–10^4^ m) tracks of presumably low-sinuous or straight channels. By the extent/thickness ratio, the composite form of members should be considered as a more or less segmented sheet. The main features of their composition are determined by the presence of one, two or probably multiple cycles together with the ratio between thickness of floodplain and channel facies within cycles. In the lower cycles of accretionary members, the floodplain facies are preserved rarely or absent, being eroded. The channel constituent can also be reduced in this case.

The seventh order surfaces define regionally extensive, separating sequences or the base of the incised valley. These sequences are not found in the present case, on the contrary, a multiplicity of disjunct (vertically and laterally) members, which, however, may have certain unity and conditional separation surfaces of this order. In search of such unity, the spatial position (valley reach, altitudinal range), specific association and facies as well as the source provenance of the alluvial clasts and grains (§4.1) have been compared (table 2). This provided an opportunity to distinguish several macro-units and interpret them in terms of the fluvial style.

**Table 2.** Comparison of the sedimentary units by: altitudinal range, association, stream morpho-sedimentary style and clasts-grains provenance. (a.w.l, above water level; b.w.l., below water level.)

formation, Reach, Unit	altitudinal range (in metres)	succession	facies	stream morpho-sedimentary style	clasts-grains provenance
conglomeratic Fm.	20−50 a.w.l	—	Gmm, Gcm, Fsm	gravel (mud, sand) non-channelized hyperconcentrated and debris flows	Carpathian
1.1	20 a.w.l–16 b.w.l	—	—	sand-gravel, braided, constrained- meandering	Carpathian
2.1	170−260 a.w.l.	GS1	—	sand-gravel, meandering	Carpathian
2.2	170−83 a.w.l.	GS2	—	gravel-sand non-channelized hyperconcentrated flows	Carpathian, Dniester
2.3	230−190 a.w.l.	Sh		sand, shallow	—
2.4	190−62 a.w.l	S3	Gh, St	sand-gravel meandering	Carpathian, Dniester
2.5	80 a.w.l.–16 b.w.l.	G2, G4, GS2	—	gravel constrained-meandering	Carpathian, Dniester
Balta Fm. (upper unit)	180−250 a.w.l.	—	St, Sp, Sh, Sr, F	sand free-meandering	Ukrainian Shield, Podillia
3.1	200−150 a.w.l.	S4	—	sand free-meandering	Podillia
3.2	154−90 a.w.l.	S1, GS3	Fl, Sl, Sp	sand-gravel meandering	Podillia, Carpathian, Dniester
3.3	128−0 a.w.l.	G1	Gm, Gcm	gravel constrained-meandering, gravel hyperconcentrated and debris flows	Podillia, Carpathian, Dniester
4.1	145−85 to 14 a.w.l.	S4, S5,	St, Sp, Fl, Fsm,	sand free-meandering, braided	Podillia, Carpathian
4.2	85−45 to 0 a.w.l	S3	Gh, Gcm	sand-gravel constrained- meandering	Carpathian, Dniester
4.3	20−0 a.w.l	G3, GS2	Sh, St, F	sand-gravel constrained- and free-meandering, mud-anastomosing,	Carpathian, Dniester
Dniester Liman Fm.	23 a.s.l.–72 b.s.l.	—	St	sand free-meandering, down- and backfilling	—
Roksolany Fm.	72 a.s.l.–5 b.s.l.	—	—	—	—

Each macro-unit is designated by number, e.g. Unit 2.1 consisting of the reach number (first digit) and the number of units (second digit). The separate members are also distinguished within some of the macro-units. Their number consists of three digits (e.g. Member 2.1.2), where the third digit designates a proper member. In order to correctly determine the relative age of units and members, it should be remembered that in a degrading valley, each underlying element is younger than the overlying one in cross-profile. However, in the near-mouth valley segment, where there is a series of superimposed members, this order is reversed. The same occurs for some accreted members in the degrading valley part.

The detailed description of the units and fluvial formations (they were of other origin than the Dniester valley, but were integral components of the common fluvial evolution) within each valley reach and reach’s segments is represented in sub-sections below.

#### 5.3.2. Reach 1

**Figure 7.**
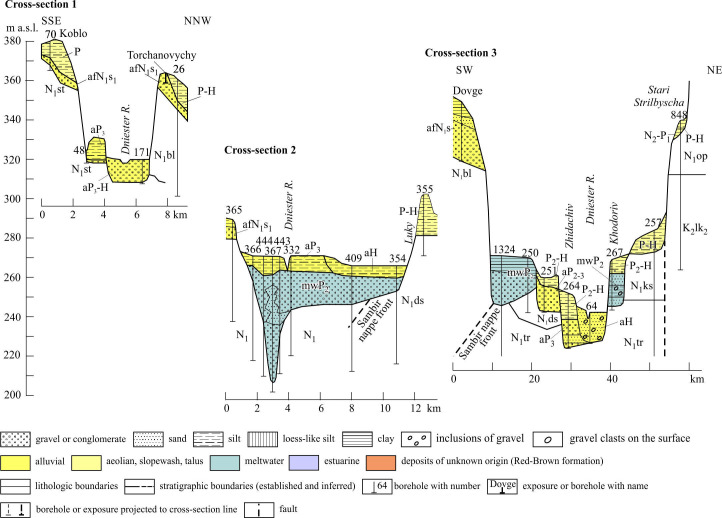
Cross-sections of the Dniester valley (Reach 1): cross-sections 1 and 3 based on archive data; cross-section 2 according to [76], modified. Stratigraphical indices: K_2_, Upper Cretaceous; N_1_, Lower Miocene; N_2_–P_1_, Pliocene–Lower Pleistocene; P–H, Pleistocene–Holocene; P_2_–H, Middle Pleistocene–Holocene. Indices of regional stages: s_1_, Lower Sarmatian. Indices of suites in order from younger to older: ds, Dashava; ks, Kosiv; tr, Tyras; op, Opillia; bl, Balychy; st, Stebnyk; lk, Upper Lukvyn. ‘Regional Stage’ and ‘Suite’, terms used in ex-USSR countries, corresponded as a whole to international terms ‘Stage, Substage or their part’ and ‘Formation’ respectively. Sedimentary environment: a, alluvial; af, alluvial fan; mw, meltwater; sw, slopewash; t, talus; e, aeolian; es, estuarine. For locations see figure 1b.

As a consequence of the almost complete absence of outcrops and small number of boreholes, the characteristics of the alluvial units of the valley are extremely scarce here. There are two obvious examples: the uppermost unit represented by Conglomeratic Formation and lowest Unit 1.1 of the ‘proper’ Dniester valley separated by an erosional slope 20−50 m high (figure 7).

According to [41], the Conglomeratic Formation is represented by scattered outliers capping the piedmont interfluves (500–300 m a.s.l.) in a strip up to 40 km wide. This overlies more ancient Miocene molasses as well as different units of the platform sedimentary cover under the common plane inclined towards the platform. The formation proper is composed of conglomerates, muds and sands, from 3−4 to 44 m thick. These deposits include Gmm, Gcm and Fsm facies (in the classification [14]), as well as associations of debris and mud flows. The conglomerates also include solitary fine-medium boulders.

Unit 1.1 combines the lower members of Reach 1, the thickness of which ranges from 9 to 20 m. The member of the first above-floodplain terrace includes channel pebbles and overlying floodplain silts in places. Its base is below the base of the modern channel. The modern member of the floodplain terrace is largely represented by pebbles, among which medium and large pebbles dominate; the content of the sand matrix ranges from 20 to 32% [34].

It is only possible to indicate that the youngest members of Reach 1 belong to gravel-bed rivers. As for the modern Dniester, it demonstrates braided style at the transversal part and mixed braided-meandering style within the longitudinal part.

In this segment about 9 km long between transverse and lengthwise parts of the valley, its base is termed the ‘Upper Dniester Swamps’ (figure 1b and 7, cross-section 2). The thin alluvial deposits of the two youngest members consist predominantly of vertically accreted mud covered by peat (low-energy, cohesive floodplain). In turn, they are underlain by pebbles and sands, 5 to 36 m thick [76]. The sand-gravel deposits infill the incisions which have uneven outlines in plan, and are absent both up and down the Dniester valley. The structure of this area has no clear explanation in hydrology nor tectonics. Demediuk & Sokurov [76] suggested, without supporting evidence, that these incisions are infilled karst sinkholes.

At the same time, it should be noted that this area is located near the maximum extent of the Middle Pleistocene glaciation (Sanian 1 and 2 [38]), and 14−16 km to the southeast of borehole no. 145, where glacial deposits were found [25]. In this borehole, under a thick layer of till, there are meltwater sands with pebbles up to 11 m thick, which can be correlated with the incisions of the Upper Dniester Swamps. The latter has two parts: the upper, which occupies the entire bottom of the valley, and the lower, a V-shaped narrow one. The morphology and variable composition of the deposits of the lower incisions are similar to the incisions and filling deposits of the tunnel channels described in the area of the Dnieper and Oka glaciations in Ukraine [77]. The upper part of the strata indicated may be associated with the valley sandur, the deposits of which are also identified (figure 7, cross-section 3).

#### 5.3.3. Reach 2

**Figure 8.**
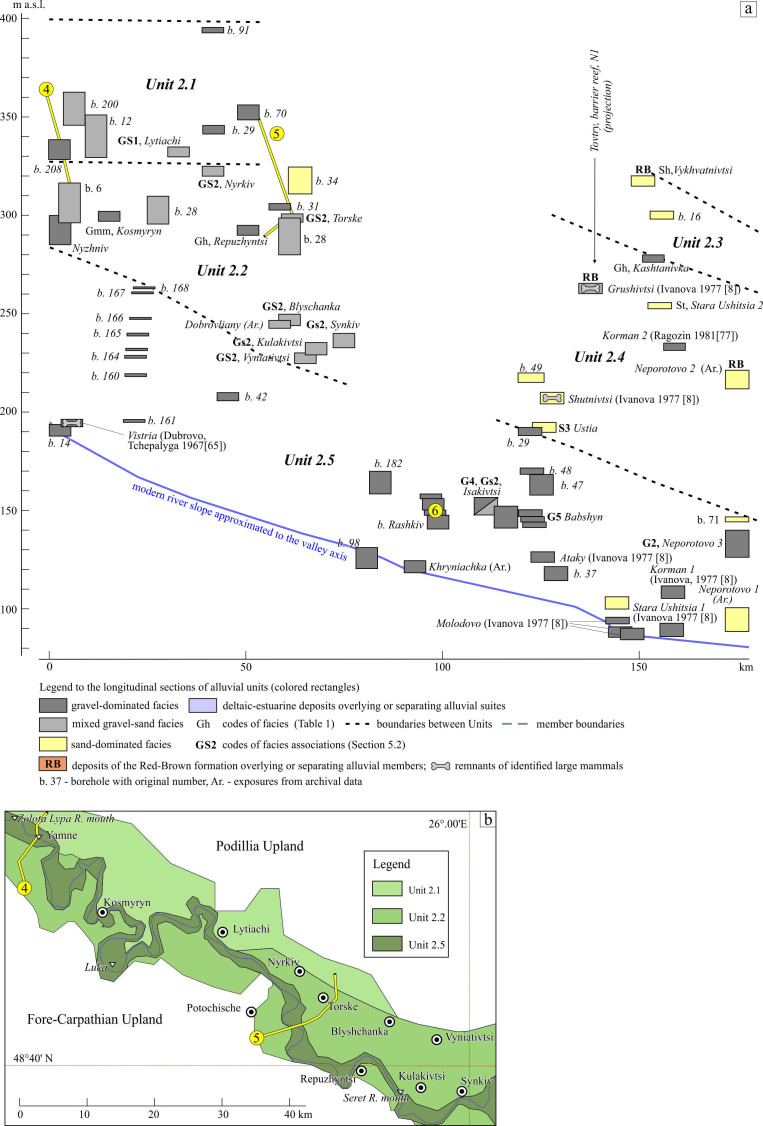
Reach 2: (a) longitudinal section of alluvial units, the site Korman 2 is cited according to [78]; (b) map of the macro-units (upstream segment of Reach 2). See location including cross-sections in figure 1c.

**Figure 9.**
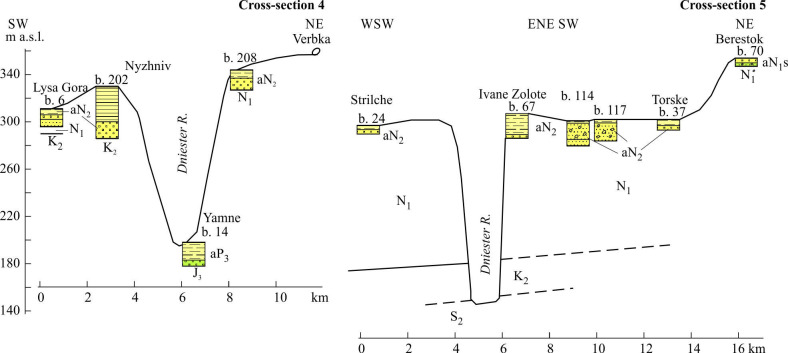
Cross-sections across the Dniester valley (Reach 2, upstream segment). Stratigraphical indices: J_3_, Upper Jurassic; S_2_, Upper Silurian. International indices (age supposed): N_2_, Pliocene; P_3_, Upper Pleistocene. See location in figure 1c, legend and other indices, in figure 7.

The members of Reach 2 are grouped into several units (figures 8 and 9) which essentially differ by their lithofacies characteristics between up-valley and down-valley disjunct segments, except for the lowest unit. The significant gaps in the distribution of alluvial deposits between these areas as well as east of the longitude of the Stara Ushitsia along the left flank of the valley are noteworthy, making the position of the valley’s boundary quite indistinct there.

Uppermost Unit 2.1 (260–170 m a.w.l.) occupies a small area within the upper left slope between the Zolota Lypa and Seret rivers. Its lower boundary is almost horizontal. In some places, the solitary pebbles of the Carpathian origin are recorded 10 to 20 m lower than the plateau summit level. The alluvial deposits of Unit 2.1 (up to 21 m thick) are referred to the gravel-sand association GS1. This gravel is of Carpathian provenance, one of the coarsest and most rounded in the valley units. This association points to channelized high-velocity deposition in gravel-sand meandering stream with depth not less than 2.0−2.5 m, subjected to an unstable flood regime. An absence of floodplain facies is evidence of an abrupt water-level fall or erosion of these facies during extreme events. Such fluvial style is closest to the style of the alluvial fans of the Conglomeratic Formation of Reach 1 [41].

The members of Unit 2.2 (between the Zolota Lypa and Seret rivers) occupy a wide altitudinal range: 170−83 m a.w.l. The lowest of these members shows obvious decline down-valley. It is encountered on both valley flanks, but mostly on the left side, being composed of alluvial deposits (2–20 m thick) referred to the very monotonous two-component GS2 association with characteristic diffuse stratification inherent to the hyperconcentrated and probably non-channelized gravel-sand streams.

As in the upper unit, the Carpathian material (red and brownish-grey jasperoids, milky quartz and fine-grained sandstone) dominates here, but with the addition of local rocks, in particular black flints derived from the Cretaceous strata. Less commonly, at the lower contact with the Miocene limestones, angular, flattened and elongated fragments of this rock are found. In places they form a calcareous breccia. The opaque minerals (more than 50% of content) followed by garnet make up the mineral assemblage of the sands’ heavy fraction (§4.1).

Unit 2.3 occupies the uppermost position (230–190 m a.w.l.) in the downvalley part of the Reach 2 but essentially differs from Unit 2.1. by the sandy channel facies (Sh) including granules and fine pebbles. Such facies usually originated in shallow mid-energy streams. It is overlain by clays of the Red-Brown Formation in some places.

Unit 2.4 occupies the valley’s macro-slope between the Seret and Kalius rivers in wide altitudinal range: 190−60 m a.w.l. It includes a set of gravel- and sand-dominated (Gh, St) facies, including S3 association (1.5−6.0 m thick). In comparison with the up-valley’s intermediate Unit 2.2 its gravel constituent is finer. Some of the members are covered by deposits of the Red-Brown Formation. The members of Unit 2.4 are referred to the shallow sand- and gravel-dominated meandering streams with more or less developed floodplain sedimentation.

Lowest Unit 2.5 in the interval 80−60 m a.w.l. up to 16 m below water level (b.w.l.). is characterized by gravel-dominated facies. There are two most pronounced varieties of them. One is thin (2–5 m) one-cycle degrading members composed of gravel-dominated channel and sand-mud floodplain facies (G5). They belong to typical canyon gravel-bed shallow meandering rivers with relatively regular flood regimes. The other consists of thick (8–13 m), one-/two-cycled or probably multi-cycled members with a wider set of channel facies, referring to sand and mixed associations. The floodplains of both varieties refer to the high-energy, laterally migrated scrolled type.

Ivanova [8] also pointed to the existence of deepening of the base of the first terrace suite below river level within Reach 2. Near the Yamne (figure 8a) the riverbed pebbles were encountered by a borehole at a depth of 16 m below the modern water level owing to deep entrenchment with subsequent accretionary infill. On the other hand, in some places, the bottom of the modern channel is represented by bedrock, lacking any alluvial cover material.

#### 5.3.4. Reach 3

Until recently, the Kuchurgan Suite occupied the uppermost position in the terrace staircase of the Diester valley (Reaches 3 and 4) and the overlying Balta series [12]. However, following the new thorough lithofacies analysis, this suite was included in the deltaic Balta Formation as a thick member of its upper sand-dominated delta plain [44]. It consists of a basal ‘lag’, St, Sp, Sh facies (according to the present codes) in the lower part of the section, and Sr and F facies (sometimes very thick) in the upper part but with different proportions.

The heavy mineral assemblages of sands refer to the Ukrainian Shield and Podillia provenances. There are so-called ‘Carpathian Pebbles’, mud balls, intraformational sandstone clasts, nodules and debris of these materials. The upper unit of the Balta Formation is accretional, reaching 40−70 m thick. Its fluvial style (especially uppermost members) can be defined as associated with non-confined or free-meandering sandy streams with medium-energy, non-cohesive, laterally migrated floodplains.

**Figure 10.**
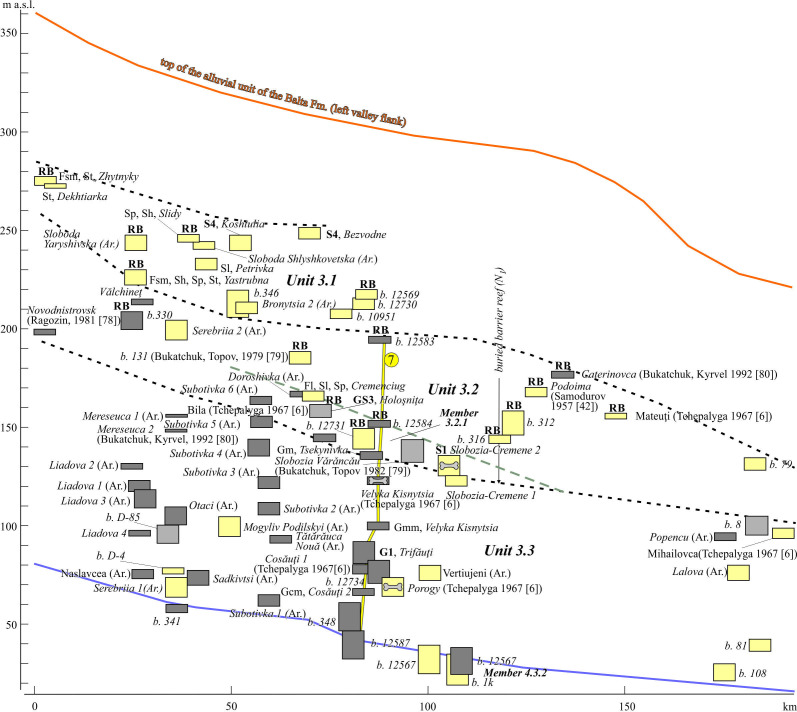
Reach 3, longitudinal section of alluvial units. See location including cross-sections in figure 1d, legend and other indices, in figure 7. The borehole 131, sites Slobozia Vǎrǎncǎu are cited according to [79]; sites Mereseuca 2 and Caterinovca are cited according to [80].

**Figure 11.**
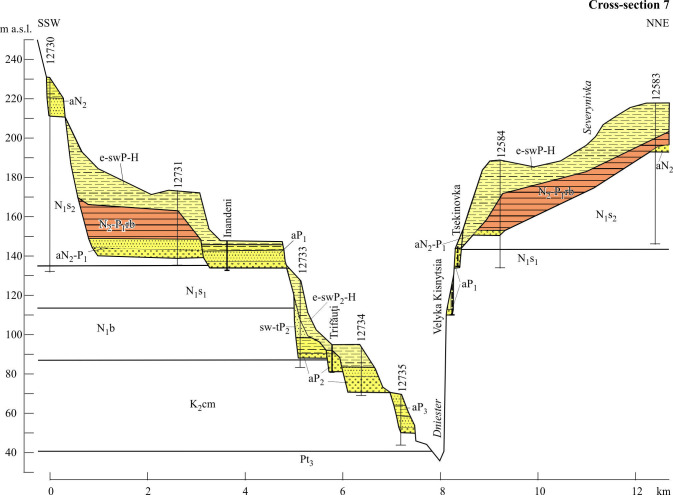
Cross-section of the Dniester valley (Reach 3). Stratigraphical indices: cm, Cenomaian; Pt_3_, Neoptorozoic. Indices of regional stages: s_2_, Middle Sarmatian; b, Badenian; rb, Red-Brown Formation; P_1_, Lower Pleistocene; P2, Middle Pleistocene. See location in figure 1d, legend and other indices, in figures 7and 9.

Three units of alluvial members of the proper Dniester valley are distinguished in Reach 3 (figures 10 and 11). The two upper units as well as Balta Formation are overlain by deposits of the Red-Brown Formation in many places.

Unit 3.1 lies 50−70 m below the top of the Balta Formation being incised in its strata. It combines scattered outliers within local interfluves in the altitudinal range: 200−150 m a.w.l. They are 2−13 m thick dominated by various channel facies of S4 association. The zircon–garnet–rutile Balta’s assemblage of heavy fraction is replaced here by garnet-leucoxene-ilmenite assemblage, but with continued predominance of ‘Carpathian Pebbles’, quartz grains and nodules in the gravel. In accordance with the facies association, the deposits of Unit 3.1 are referred to the sandy meandering fluvial style with medium-energy and non-cohesive floodplains.

Unit 3.2 occupies an intermediate position in the range 154−90 m a.w.l. It is represented by a gravelly sand-dominated facies (Fl, Sl, Sp) and S1 association. The lower members of this unit include sand-gravel facies and gravel-dominated facies. The members of Unit 3.2 are 3−10 m thick, but in the area upstream crossing the ancient reef zone, their thickness increases to 23−26 m, including superimposed members. In some places, the channel deposits are overlain by 12−14 m blanket of floodplain muds [79]. An assemblage of heavy fraction in sands is the same as in Unit 3.1 and retains in Unit 3.3. Most of their features are evidence that a sandy meandering style is gradually replaced down-unit by a gravel meandering one.

On the whole, the interval of Unit 3.3 continues downvalley between the river level and 115−65 m above it. The members of this unit are often capped by loesses, their derivatives and hillslope deposits. Most of them consist of gravel-dominated facies and their composition (G1 association, Gm and Gcm facies), members thickness and interpretation of fluvial style do not differ from those of the lower unit of Reach 2.

#### 5.3.5. Reach 4

**Figure 12.**
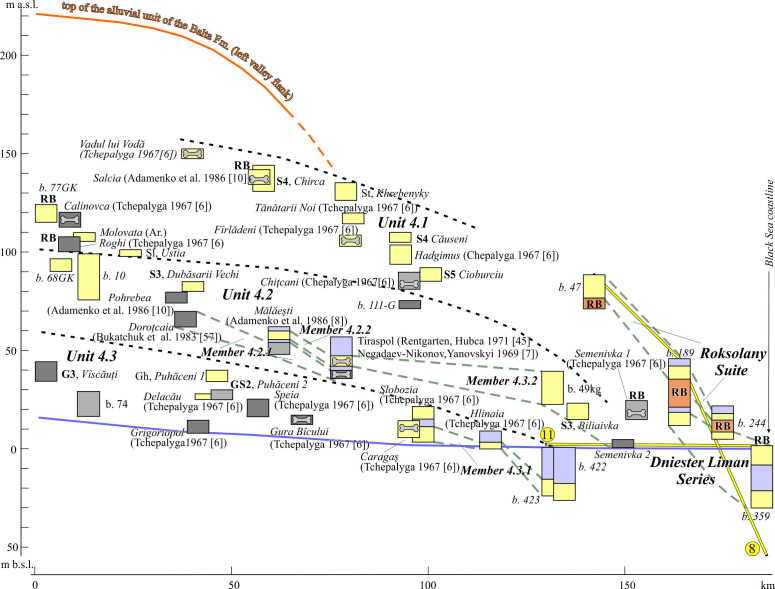
Reach 4, longitudinal section of alluvial units. See location in figure 1e, legend, in figure 7.

**Figure 13.**
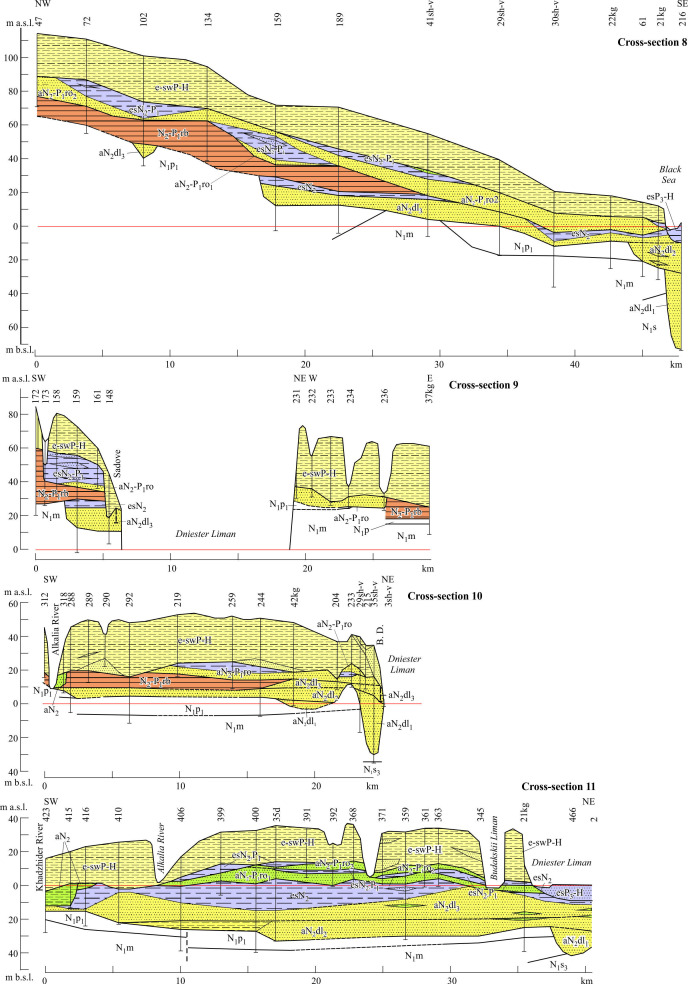
Cross-sections of the Dniester valley (Reach 4). Stratigraphical indices: s_3_, Upper Sarmatian; p_1_, Lower Pontian; m, Meotian; dl_1_, Lower Dniester Liman Series; dl_2_, Middle Dniester Liman Series; dl_3_, Upper Dniester Liman Series; ro, Roksolany Suite; B.D., Bilgorod Dnistrovskyi. See location, including cross-sections in figure 1e and figure 12; legend and other indices, in figures 7, 9 and 11.

Three main units of the alluvial deposits (4.1, 4.2 and 4.3) are distinguishable within Reach 4 (figures 12 and 13). Unit 4.1 steeply declines downvalley (Vîşcăuţi–Botna River mouth) from the 145−85 interval to 14 m a.w.l. Its members are composed predominantly of St, Sp, Fl, Fsm facies referred to the S4 and S5 associations. Their thickness varies from 3 to 12 m. Some of the members are covered by deposits of the Red-Brown Formation. The gravel-dominated and mixed facies associations are less common but occur as far as the mouth. These observations point to the dominant sand-free meandering and braided fluvial style.

According to prospecting work [46], the members of the upper Unit 4.1 are outliers stretching from hundreds of metres to 1.5 km. The same source indicates that in gravel, the fractions from medium to very coarse pebble are predominant. In the most downvalley site (Semenivka 1), the intermediate member is represented by mixed facies associations including not less than 2−3 m thick conglomerates containing clasts up to coarse pebbles [6].

Unit 4.2 occupies an intermediate position in the narrower part of the valley, retaining a degradational architecture. It starts from the altitudinal range of 85−45 m a.w.l. repeating further the inclination of Unit 4.1. This unit is represented by different groups of facies. The authors’ data, as well as interpretation of the historical lithological descriptions [6,43,45,81], are evidence that the Gh and Gcm facies typify of gravel deposits and S3 association is the most distributed among sandy deposits. Tchepalyga [6] noted the presence of boulders at the base of some members. The members’ thickness varies from 4 to 23 m. The splitting of Unit 4.2 on at least two correlated members (Units 4.2.1 and 4.2.2), separated by estuarine stratum is distinguished there. The characteristic of the estuarine deposits in the Tiraspol site ([6]; electronic supplementary material, S6) is the basic for interpretation of its analogues in other members of Reach 4.

The common occurrence of lower Unit 4.3 is similar to the lowest units of other reaches. It appears first from 20 m a.s.l. also plunging towards the sea. It shows a continuation of its neighbouring Unit 3.3 with a dominance of the gravel facies (G3 succession) on sandy (Sh, St facies) and gravel-sand (GS2 association) ones. Most members (5–12 m thick) pinch-out downstream. The gravel facies are replaced by sandy ones in this direction. At 12 km from the delta top a meandering style of the present channel changes to an anastomosed mode resulting from a declining slope. There, the channel sands of the modern member gradually transition to the floodplain mud facies of the Dniester estuary. These muds overlie the Holocene estuarine muds of the marine New Euxinian horizon [13].

### 5.4. Morphology of the valley

Concepts related to river valleys, despite their simplicity and apparent obviousness, are not always interpreted unambiguously and therefore require precise definitions. The Dniester valley is a negative, relatively narrow and elongated landform created by a permanent degrading stream, with two inclined lateral slopes. In some places, the slopes have an upper bend (margin), i.e. valley border, in others this bend is indistinct or absent, then the border is fixed by the distribution of alluvial deposits, including gravel placers in particular, or is accepted conditionally. The valley bottom is formed by the lowest flattened terrace (floodplain) with the channel. The difference between the upper boundary and the base of the lowest-lying alluvial deposits or the base of the modern channel in bedrock is its depth, and the distance between the two opposite upper borders in a direction perpendicular to the valley axis is its width.

The valley primarily originated by linear fluvial erosion, which is especially evident on the pure bedrock steep slopes. In the case of the Dniester, it is usually observed in its lower narrower part, i.e. the canyon. The slopes are complicated by secondary degraded (tributary valley, gullies) or aggraded (slopewash fans, sheets) landforms.

One of the controversial issues of the valley architecture is the river terraces identified and their number. In fact, the manifestation of terraces in the Dniester valley slopes is the exception.

**Figure 14.**
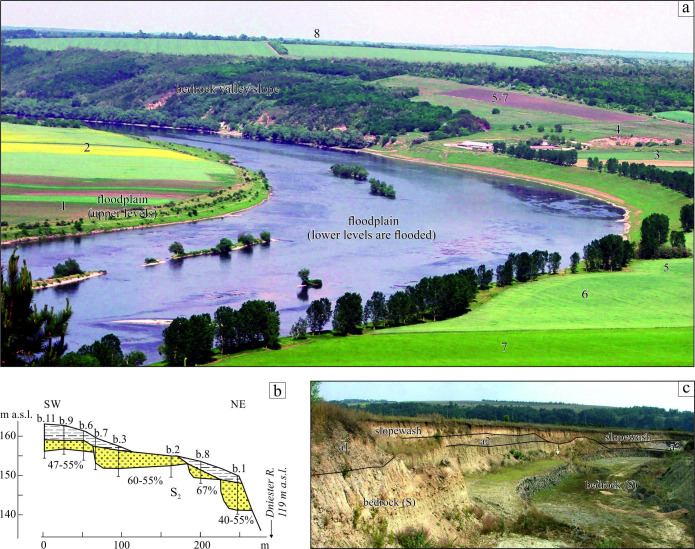
Dniester members-terraces: supposed and real. (a) Supposed terraces with conditional numbers based on conventional visual perception, Tîrgul-Vertiujeni, northward view from the right bank of the river; (b) the ladder of the alluvial members on the cross-section through the projected pit of gravel extraction (content of gravel fraction in per cent), Rashkiv; (c) three sequential alluvial members (a1, a2 and a3, the base of the a2 member – 44 m a.w.l), 1.5–3.0 m thick on the eastern wall of the quarry (maximal depth, 8−9 m), Babshyn. S, Silurian; S_2_, Upper Silurian. See location in figure 1c,d.

Indeed, there are places where terrace surfaces can be determined visually by up to a dozen in number (figure 14a). However, most often the surfaces that can confidently be recognized are only 2−3 in one cross-profile and, as a rule, the lowest ones. As will be shown below, in the supra-canyon part of the valley only 1−3 terraces can be traced reliably within some reaches, using topography. Across meander lobes in the canyon, numerous slope bends can be mapped, which may correspond to the riser-thread inflections, but as follows from the comparison with drilling data, this is not always the case. This distinction results from the active slope processes. Another powerful factor in the natural levelling of terrace risers and their burial is the accumulation of aeolian material. An unambiguous conclusion is that most of the Dniester terraces are buried landforms.

Another misconception is associated with the idea of a fixed and limited number of terraces. As mentioned in §4, this confusion arises from the analysis of published data, however, there are sufficient other reasons to reconsider these theses. Drilling the slope of the meander lobe near the Luka (figure 1c) with a step 0.6−0.7 km between boreholes, revealed seven alluvial units in the altitudinal range of 90 m from the valley bottom. The exploration boreholes of the deposit of a sand-gravel mixture near the Rashkiv (Reach 2, in the middle part of the canyon slope; figure 1c) revealed on a gentle slope (dip angle: 3.4°, length: 250 m, altitudinal range: 25 m) four members. The height difference between their bases is 3−7 m, and the width of the terraces reaches 30−110 m.

The next example is a quarry near the Babshyn (Reach 2; figure 1c), cut along the slope of the meander lobe (dip angle: 4.4°, length: 90 m, range: 7 m). Three alluvial units with clear boundaries are identified here as a continuous stairway (figure 7c). If the height difference in the unit’s soles of about 4 m is taken as a basis, it can be assumed that about 38 of them could have formed in the canyon part of the valley (150 m range). This should be compared to the 14 terraces previously proposed (§4) for a valley up to 250 m deep.

Figures 7–14 demonstrate a plethora of alluvial units occurring at different altitudes within neighbouring valley segments. It is obvious that many, especially the most ancient units, have not been preserved; others have not been discovered, but one thing is certain, the primary number of units-terraces is several times greater than what was only recently postulated.

The detailed morphology of the valley’s reaches is represented in the electronic supplementary material, S1, while their main morphological parameters are summarized in table 3.

**Table 3.** Morphological parameters of the valley and canyon within reaches and their segments.

Reach (number)	segment	valley			canyon
		width spacing (km)	depth spacing (m)	mean direction (azimuth in degrees downstream)	canyon sinuosity (average)
1	mountains output—Stryvigor R. mouth	2−6^a^	56−24^a^	62	—
	Stryvigor R. mouth— Zolota Lypa R. mouth	2−21	24−180^a^	123	—
2	Zolota Lypa R. mouth—Seret R. mouth	3−20	180−243	120	2.3
	Seret R. mouth— Kalius R. mouth	6−22	138−232	88	2.1
3	Kalius R. mouth— Camenca R. mouth	29−7^a^	197−104^a^	128	3.6
	Camenca R. mouth— south end of Reach 3	7−13^a^	101−104	154	1.1
4	northern start of Reach 4—Botna R. mouth	13−43^a^	104−145^a^	159	1.7
	Botna R. mouth— coast of the Black Sea	21−53	145−67^a^	145	—

^a^ Spacing indicates downstream trend. The dash in column ‘canyon’ means absence of this geomorphic element in the corresponding segment of the valley.

**Figure 15.**
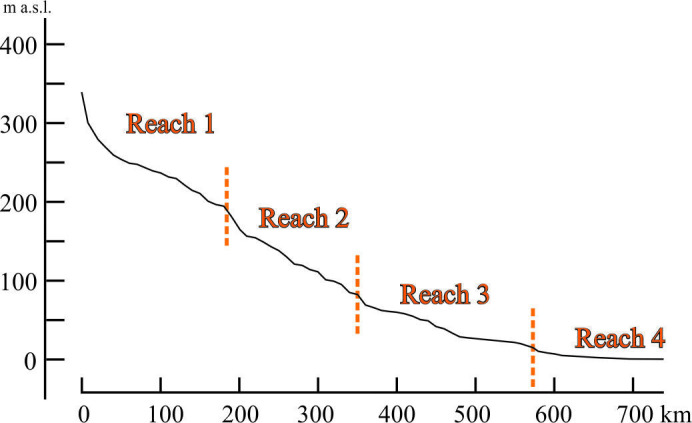
Modern longitudinal profile of the Dniester River (plain reaches). The gradient downstream the knickpoints between Reaches 1 and 2 increases five times, Reaches 2 and 3 increases 2.7 times and Reaches 3 and 4 increases 3.6 times.

An important output of the ‘terrace problem’ analysis is evidence of the incorrectness of the long-distance pure altitudinal correlation (§4.3), as well as compilation and comparison of the widely adopted into practice plots of longitudinal profiles of different ages in the absence of sufficient numbers of independent and reliable dating. However, the single profile of the modern Dniester River (figure 15) unexpectedly turned out to be associated with the boundaries of the valley reaches, identified according to other morphological and lithofacies criteria. It has different forms in reaches: concave–convex (Reach 1), generally straight, but with numerous secondary small convexities (Reaches 2 and 3) and smoothly concave-graded (Reach 4). The boundaries of reaches coincide with the most abrupt knickpoints. The slope of the profile increases fivefold at the Reaches 1/2 boundary, 2.7 times at the Reaches 2/3 boundary and 3.6 times at the Reaches 3/4 boundary. None of them is associated with changes in river’s water/solid load discharge and rocks erodibility or channel damming. According to Demoulin *et al.* [15], the other probable causes of these knickpoints are regional tectonics or/and large-scale upstream propagation of an erosion wave.

## 6. Discussion

### 6.1. Justification of the stratigraphical scheme

A critical collation of previous results and new data made it necessary to clarify and, in some aspects, revise the provisions of the previous approach to the stratigraphy of the Dniester valley. The new treatment involves sequential consideration of stratigraphical criteria in order of priority, including the general age limits by means of marker beds (already considered in §4.3 and accepted in the present study), updated direct age determinations of alluvial sequences using fossils, correlation of macro-units distinguished by lithofacies criteria, correlation with regional and continental-scale events, geomorphological correlations and their results.

#### 6.1.1. Direct age determinations by remains of vertebrates

The stratigraphical priority in this case is given to the remains of terrestrial mammals from established locations, distinguished to level of species and clear taxonomy (electronic supplementary material, S7). The selection principles restricted the number of find-sites to 29. These finds belong to the following mammal families: Elephantidae, Mammutidae, Equidae, Cervidae, Rhinocerotidae, Ursidae and Felidae, included in European Mammal Zones MN13–MN17 up to the present. Only those index-representatives are considered which appearance, living frame or extinction falls within the studied timespan. The up-to-date estimates of species living intervals with their presence in European domains were used [82–89]. The seven sites that yielded small mammals (*Leporidae* and *Cricetidae* families) essentially complement the noted scheme in the summary and evolutionary interpretation [68].

Juxtaposing the living intervals of different species in the same or close locations, narrower age frameworks for stratigraphic division could be established. It is important that palaeontologists usually connect these extant periods with boundaries of the standard stages or their main spans, which determines the accuracy of timescale (10^4^–10^6^ yr.). To this should be added a previous review on terrestrial vertebrate fossils found in the Balta Formation belonging to MN9–MN13, which suggests an approximate 11.2 to 5.3 Myr age range [44]. Together with updated and clarified data on the Dniester valley, they form an independent foundation for the present stratigraphical scheme (figure 2).

#### 6.1.2. Altitudinal and lithofacies correlation

**Figure 16.**
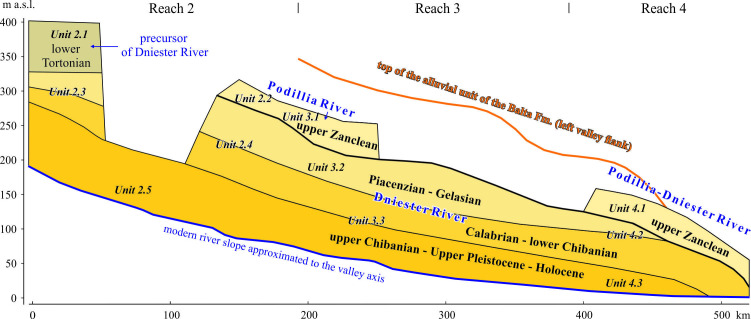
Integral longitudinal stratigraphical profile through Dniester Reaches 2, 3 and 4.

The errors of the previously proposed correlations are discussed in §§4.3 and 5.4. At the same time, the number of qualified palaeontological age determinations is limited. As shown below, tectonic movements, sea-level fluctuations and general changes in the palaeogeographical situation could and indeed did lead to distortion of idealized concepts, including equilibrium and topographical ambiguity of the members’ allocation within reaches and their segments. Also, the facies and their associations can be naturally replaced or pinched out downstream in parallel with the vertical profile in response to temporary and spatial changes of the gradient, river discharge and solid load. Based on this evidence, the authors followed an alternative path of a less ‘detailed’ correlation, not of members-terraces, but of macro-units (§5.3) between valley reaches (figure 16). This correlation is based on facies similarity, relative elevation, fluvial style and clasts-grains provenance taking into account other stratigraphical results.

The units of the Conglomeratic Formation and uppermost Unit 2.1 are very close in their uppermost position and facies. This is not a temporal but a spatial relationship, when the subsequent process was the next stage of the previous one. Units 2.2, 3.1 and 4.1 also show a high level of kinship taking the uppermost position in Reaches 2 (downstream part), 3 and 4. However, they differ in their assemblage composition of the heavy mineral fraction. The composition of alluvial deposits of Unit 3.1 implies a continued significant influence of the Podillia provenance. Therefore, this erstwhile stream is called here the ‘Podillia River’. This could be the case if the river merges with that from the Carpathians between the districts of the units noted.

The lowest units (1.1, 2.5, 3.3, 4.3) are most reliably correlated. The alluvial formations associated with incisions near the Dniester mouth (Dniester Liman Series and Roksolany Suite) also demonstrate good correlation by boreholes at short distance (figure 14).

#### 6.1.3. Event stratigraphy

The application of marker beds is closely related to the event stratigraphical approach. In this case that means regional or continental-scale relatively short geological events which directly or indirectly could significantly affect the valley development. The originating event was the appearance of the Carpathian proto-land and the closure of the last Paratethys basin from the area of Reach 1 and partly Reach 2 in the Early Sarmatian [90]. This enabled the river network to develop. The next event was an arrival of the axial fluvial system in the form of the deltaic Balta Formation (Middle Sarmatian – Early Pontian [23]).

The Dniester Liman Series of Reach 4 is associated with incision in the Odessian limestones being overlain by clays of the Red-Brown Formation. This incision, restricted by the coastal region, could be related to substantial and/or abrupt sea-level fall. To date, several episodes of sea-level fall in the Black Sea basin have occurred since the lower Pontian. One of which, the greatest, is the Messinian (Portapherian) short-termed sea-level fall of as much as 500−600 m which was first identified in the Ciscaucasia part of the basin [91] and within the Romanian shelf and slope [92–94]. This is very probably a correlative to the Dniester Liman Series incision noted above, as well as for the deeply seated Near-Danube Formation (lower reaches of the Prut and Danube rivers [54]).

The next shallower sea-level fall is recorded around the Miocene–Pliocene boundary by the regional unconformity with canyons and small channels in the same part of the Romanian shelf [94]. This could be equated to the upper members of the Dniester Liman Series.

Although most of the valley occurred in the extraglacial zone of the Pleistocene continental glaciations, there was both a direct and indirect impact of the latter. The deposits and landforms of the Middle Pleistocene glaciation ( = Oka, Sanian, Elsterian) in Reach 1 are established (§5.3.2). It can be assumed that their analogues in Reach 4 are probably the alluvial deposits in the Tiraspol section forming the substrate of the widest terrace in the valley. Its Middle Pleistocene age is distinguished by mammal remains. This and some other sections included one-two units of the estuarine deposits (S6). They can be referred to the Middle Pleistocene Palaeo-Euxinian transgression of the Black Sea previously documented in the lower reaches of the Danube and Prut [9,95,96].

The youngest near-mouth overdeepening is infilled by deposits of the Ochakiv-Ant Suite. Earlier such incisions were recognized in most estuaries and in many offshore boreholes and valleys between the Dnieper and Danube rivers [97]. This has yielded ^14^C ages from 24 to 13 ka, i.e. encompassing the Last Glacial Maximum during which sea-level fell (80–120 m b.s.l.) to the margin of the Black Sea shelf.

The appearance of overdeepening within the floodplain and first terrace of the Dniester recorded in Reaches 1, 2 and 3 points to a noticeable increase of the stream discharge. A rational explanation for this could be mountain glaciation in the Carpathians, changing the river regime and/or fast melting of glaciers. The age of mountain glaciation was established recently by surface exposure dating as Younger Dryas Stadial, i.e. 12.4 ± 0.3 to 12.9 ± 0.3 10Be ka by Rinterknecht *et al.* [26]. In the condition of narrow channels sandwiched in the resistant rocks, intense downcutting was a single result of the amplified stream power.

#### 6.1.4. Geomorphological–lithological, borehole-to-borehole or exposure-to-exposure correlations

Geomorphological–lithological correlations also occur, but are secondary, locally related to several selective terraces (alluvial members) which are represented by comparably wide (10^2^–10^3^ m) fragments traced within some reaches or their large parts. Initially, this was done for the over-canyon terraces of the central part of Reach 3 [56,98]. Such terraces are encountered at the bases of the unit complexes as flat or slightly sloping surfaces of very close (not more than 10−15 m) relative height, even taking into account their covering of loess or slopewash materials in some cases. Naturally, the floodplain terrace as well as the first terrace above it are the most reliable features throughout the valley forming geomorphic correlates. These geomorphological correlations are successful where there are sufficient borings and/or exposures within short segments of reaches with close altitudinal position. They are associated with the lowest members of the Piacenzian—Gelasian complex in the central part of Reach 3 (Member 3.2.1) and the lowest members of the Calabrian—lower Chibanian complex (Early to Middle Pleistocene) within Units 2.3 and 2.4 of Reach 2 (Nyzhniv, Blyschanka, Synkiv, Kulakivtsi, Vyniativtsi, Ustia).

The same type of correlation is applicable for the Roksolany Suite and the upper part of the Porat Formation with the same altitudinal occurrence and alternations with estuarine members (lower reaches of the Prut and Danube rivers [54]).

### 6.2. Integrated stratigraphy

The integrated outputs of the approaches noted above give a more approximate yet more realistic picture (figures 2 and 16) concerning part of Reach 2 as well as Reach 3 and Reach 4. All the palaeontological dates obtained fall into the Late Pliocene–Quaternary age interval. This is the part of the scheme for which the most evidence is available. It includes three complexes with units correlated to the Piacenzian–Gelasian, Calabrian–lower Chibanian, upper Chibanian–Upper Pleistocene–Holocene. They can be cautiously considered as the major informal alluvial formations of the Dniester valley, reflecting the main phases of the valley evolution. Above them lie two more ancient complexes which do not have their own direct stratigraphical indices, but they are distinguished by their altitudinal occurrence and lithofacies characteristics. This is supposedly the lower Tortonian complex (Reach 2) close to the alluvial fans of Reach 1 and the upper Zanclean complex of Reaches 2, 3 and 4. The alluvial deposits of the three lowest members of Reach 1 (§5.5) are referred to the Upper Pleistocene Subseries and the Holocene Series. The most probable age estimates and correlations are associated with overdeepenings of the near-mouth area (Dniester Liman Series and Roksolany Suite).

### 6.3. Evolution of the Dniester valley and associated fluvial systems

**Figure 17.**
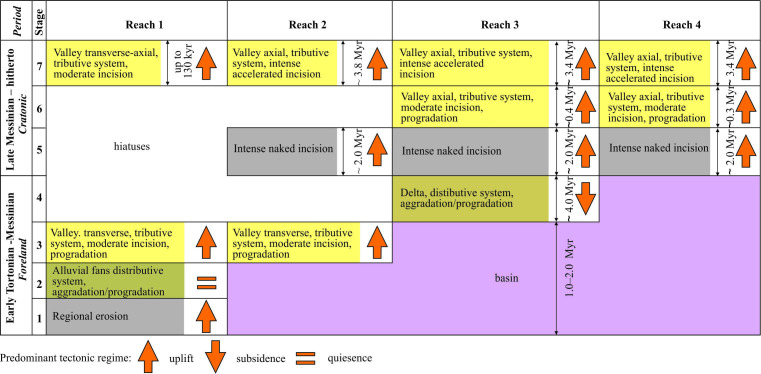
Dniester and associated fluvial systems evolution (for details see the text).

The development of the Dniester valley is treated in 10^4^−10^7^ yr. timescale in close relation to its changeable basin form (figure 17). Among the different common characteristics, the following terms imply its morpho-sedimentary reconstruction: fluvial system organization (tributary/distributary) and dynamics (degradation/aggradation, progradation/retrogradation). An interpretive contribution to the study attempts to assess key factors participating in transformation of the fluvial system (tectonics, topography, climate, rocks erodibility, base-level oscillations) as well as to establish general trends and turning points in development. Based upon the latter, the authors’ concept of the Dniester and associated fluvial systems history includes two main periods: early Tortonian to Messinian and Messinian to the present time. According to the different styles of tectonic movements, the first can be called ‘foreland’ and the second ‘cratonic’. These intervals are divided into seven stages by combination of the main development characteristics relating to individual reaches or several of them. The development of the valley during the conditions of the platform margin requires special consideration.

#### 6.3.1. Early Tortonian–Messinian, foreland period

*Fluvial development*. The retreat of the Early Sarmatian basin from the area of the Dniester Reach 1 implies the potential appearance of the fluvial–terrestrial conditions. None of Dniester’s studies, considering river activity in its basin, have taken this circumstance into account. Previously, the river’s history is dated to the Pliocene Epoch (§4.4), leaving 5−6 Myr as a ‘blank period’ or period of ‘traceless total erosion’. Nevertheless, there are several serious pieces of evidence which can be associated with fluvial processes during early Tortonian times. They include the regional erosional surface, alluvial fans and alluvial deposits of Unit 2.1.

**Figure 18.**
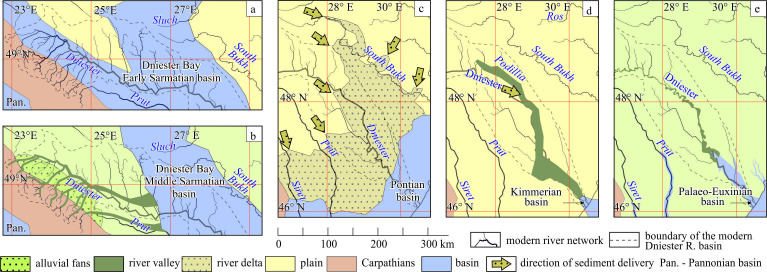
Palaeogeographical maps of the Dniester valley and associated alluvial formations: (a) Early Sarmatian (late Serravalian), situation preceding the origin of the fluvial system; (b) early Middle Sarmatian (early Tortonian), situation at the end of stage 3; (c) Meotian–Early Pontian (middle Messinian), situation at the end of stage 4; (d) Kimmerian (early-middle Zanclean), situation at the start of stage 6; and (e) Palaeo-Euxinian (middle Chibanian), episodic situation at the end of stage 7, very close to the modern one, exсept Reach 4.

Moliavko [90], based on distribution of the corresponding deposits, compiled a palaeogeographical map of the Early Sarmatian basin, including its Dniester Bay (Galiczian Gulf [22]), the latter where the Dniester valley Reaches 1 and 2 occur (figure 18a). It is obvious that the first erosional landforms were associated with the development of the Carpathian proto-land.

As the basin left the northern sector of the foreland, the most ancient post-collisional erosion incised the Miocene and nappes as well as different units of the platform sedimentary cover under the common plain being inclined towards the platform [21,41,99]. This angular erosional unconformity is shown in all cross-sections through the wedge-top zone of the orogen, including the Dniester’s Reach 1 [60,100]. This was the first manifestation of the regional ‘naked’ (or blank) entrenchment (Stage 1). The word ‘naked’ is used for a description of quite rapid incision, dominated by downcutting, during which the alluvial members, if formed, were quite local and thin. Such incision is considered opposed to the slower and more balanced process imprinted by the staircase of the alluvial members.

Since that time and onwards within Reach 1 the traces of significant restructuring of the river network are unknown, this hypothetical primary stream could coincide with the modern longitudinal position of the Dniester valley.

During Stage 2 the alluvial fans (Conglomeratic Formation) were emplaced on the previously eroded surface, creating the first distributive fluvial system in this area [41]. Teisseyre [101] included the deposits of the present alluvial Unit 2.1 within the Podillia Upland into his uppermost alluvial ‘Loieva Level’. Based on the current view, it correlates with alluvial fans as subsequent to Stage 3 of the fluvial development. This Dniester River precursor, with a gentle gradient, had a catchment that linked the transverse Carpathian tributaries cutting through the already abandoned alluvial fans where the modern transverse network coincides with ancient ones (figure 18b). Unit 2.1 recorded 50−60 m entrenchment and tens of kilometres advance in an eastwards direction. Gerenchuk [102] was first to draw attention to this turn of the valley following the retreated Sarmatian Sea, away from the grain of the Carpathian structure. It is assumed that at the same time an allocation of an independent system of the neighbouring Prut River occurred and its main valley repeated the same striking bend.

During the late Middle Sarmatian Substage, the regressive trend of the basin continued and its Dniester Bay ceased to exist. The great derangement of the East Carpathian foreland basin, including its associated fluvial system, took place and was represented by a substantial hiatus in alluvial archive. These changes gave rise to the arrival of the spacious axial distributive Balta Delta (Stage 4). This delta prograded slowly through the Tortonian and early Messinian (approx. 4 Myr) with a reciprocating mechanism of the fluvial/marine sedimentation [44]. At the beginning of the Pontian time, this system occupied the entire width of the foreland (about 60 000 km^2^) accumulating 120−380 m thick deposits. This was the termination of the ‘foreland’ period (figure 18c).

*Tectonic background*. The history described above fits into concepts of the retro-arc foreland flexural tectonics [103,104]. According to [104], the fluvial processes in the sedimentary zones of the foreland in terms [105] correspond to the tectonic flexural provinces shifting along dip and strike in through time and defining the deformation pattern of flexural uplift and subsidence. It is assumed that the Dniester Bay (figure 18a) development can be interpreted as follows: western branch as a top-wedge and foredeep; peninsula as a forebulge, eastern branch as backbulge. Numerous local flexures and faults parallel to the Carpathian grain, as well as local grabens recorded in the Late Miocene strata within supposed forebulge and backbulges ([106–109] support this concept.

The foreland has experienced underfilled and overfilled phases (‘flysch’ and ‘molasse’ styles of sedimentation [110]). The second phase (this case) means domination of continental environments including the appearance of fluvial sequences separated by unconformities and restricted to one flexural province at a time. The latter is considered as a result of temporal–spatial competition between two styles of fluvial sedimentation belonging to both transverse and axial systems.

In connection to concepts [103,104], the unconformity of Stage 1 could reflect the first stage of the overfilled phase, the so-called ‘fluvial/bypass erosion’ when alluvial sedimentation concentrates at some distance laterally from the orogenic wedge in the longitudinal stream parallel to the foreland axis. They agree that the axial fluvial system was followed by a transverse phase, which was realized by alluvial fans (Stage 2) within the wedge-top and foredeep [41]. The fans originated from the gradient provided by the previous post-collisional orogenic uplift, but with a time lag after it in conditions of tectonic quiescence within the foreland. This was followed by Stage 3 during which moderate uplift in the foredeep and displacement of the depositional zones eastwards occurred, the distributive fan system transformed into a tributary one.

Stage 4 was marked by change of the perpendicular to the Carpathians west-directed depositional dip of the basin to south-southeast-directed one along with 90° rotations of facies belts [23,111]. The forebulge shifted more than 100 km eastwards within the Ukrainian Shield [23]. Catuneanu [104] explains such changes by isostatic rebound during orogenic unloading intrinsic for the overfilled phase, whereas the direction of tilt was induced by the strike variability during orogenic loading (underfilled phase). All the Balta Delta area was subjected to weak subsidence with depositional average rate –0.025−0.95 m ka^–1^ (based on thickness of deltaic deposits [44]).

*Climatic background*. Based on previous analyses [41], it seems that the reconstructions of precipitation in the area of Central and Eastern Europe for the late Serravalian time, based on palaeoecological interpretation of different groups of organisms, gave contradictory assessments; whereas, the presence of land (at least uplifted islands at the site of the Carpathians) was by itself a critical point for concentration of moisture, the role of which increased during the course of mountains’ rise. In particular, it was concluded that frequent large floods and erodible rocks provided the formation of coarse-grained alluvial fans in common. Apparently, the conditions conducive to precipitation in the Carpathians remained until the second half of the Middle Sarmatian age.

Unlike previous opinions concerning the Carpathians as the main water-sediment source for the Balta Formation [44], the present evidence points to a decrease in their role. Beginning from the end of the Middle Sarmatian age, the northern part of the foreland basin became dry land and the catchment area increased tenfold. This partly explains the fact that the influence of the Carpathian provenance on the Balta sediments is almost imperceptible. This conclusion was made on the basis of the present comparison of truly Carpathian material in the Dniester valley within Reaches 1 and 2 and that in the Balta Formation (§5.3.4). The traces of the delta-feeding streams and their deposits are not found. It can be assumed therefore that there were relatively small but numerous suppliers of predominantly sand and mud. Probably, the Dniester stream proper was only one of them. As a whole, the results of climatic interpretation of the Balta Formation indicate a relatively dry climate with a flashy, strongly seasonal discharge regime of the rivers, which has been changed to more a uniform one towards the end of the Balta delta’s existence [44].

#### 6.3.2. Late Messinian—present time, cratonic period

*Fluvial development*. The flat deltaic plain at the end of the Balta system functioning with minimal slopes was an ideal place for river derangement. It began with 50−70 m deep naked entrenchment into the Balta Formation (Stage 5). This entrenchment developed in two main directions: basinward, adapting to falling base-level and upstream aiming for a balanced longitudinal profile. At the same time, there was a redistribution of the catchment area. Five new river basins stood apart within former foreland: the Siret, Prut, Dniester and South Bugh [13] plus Podillia (data presented herein). The Dniester Reaches 3 and 4 were formed whereas Reach 2 inherited the position of the Dniester’s ancient precursor.

It is likely that the entrenchment into the Balta Formation and the Dniester Liman incision can be associated with the same great Messinian sea-level fall (§6.1.3). The build-up of the Dniester Liman Series in conditions of sea-level rise continued up to the end of the Messinian Stage.

At some point, the entrenchment in Reaches 2, 3 and 4 slowed giving more possibilities for partial alluvial accumulation (Stage 6). In the area studied, it is recorded by Units 2.2, and 3.1 of the Podillia River and Unit 4.1 (in common with one of the Dniester and Podillia rivers; figure 18d). These units were formed in the background of continuous degradation. Regarding the timing of the incisions, it can only be said that it was post-Odessian (late Messinian Substage), i.e. syn- or post-regression of the early Pontian basin, whereas accumulation of the alluvial units falls on the end of the Zanclean Stage. Between these events, there is a large hiatus.

The newest Stage 7 of the trunk Dniester River proper development occupies the Piacenzian–Pleistocene–Holocene times. During this phase, all the Dniester reaches were combined (figure 18e). It is characterized by unidirectional accelerating entrenchment/accumulation recorded by numerous clusters of alluvial members. At its start, the more active tributaries of the Dniester and South Bugh rivers captured and divided the system of the Podillia River amongst themselves.

The terrace staircases have long been used to determine rates of fluvial incision [112]. Here the units’ staircases are used as a basis for the integrated stratigraphy. The difference between these two approaches is shown in §6.1. The maximal incision (about 190 m) during this phase falls on the downstream part of Reach 2 and adjoined upstream part of Reach 3 (figure 16). Its average rate was 0.027 m ka^−1^ during the Piacenzian-Gelasian; 0.045 m ka^−1^ during the Calabrian—early Chibanian and 0.144 m ka^−1^ from the late Chibanian to the Holocene, i.e. a five-fold increase. Taking into account the cross-section at the archaeological site Molodova, its dates [8,74] and overdeepening within the floodplain and first terrace above floodplain, the incision rate during last 40−45 ka within Reach 2 was 0.07−0.09 m ka^−1^. The entrenchment was accompanied by a right-sided shift (Reaches 2 and 3), transition from the sand-gravel to gravel streams and from free-meandering to constrained-meandering style (table 3) as well as valley narrowing and steepening of its sides.

Schaller *et al.* [113] obtained very close values for catchment-wide palaeo-erosion rates from terrace deposits of the Meuse River (Europe, The Netherlands), using cosmogenic 10Be within the range of 0.025−0.035 m ka^−1^ from 1.3 to 0.7 Ma and increasing rates (around 0.08 m ka^−1^) since 0.7 Ma. According to [114], the incision of the Seine, Yonne and Somme rivers (Europe, France), using ESR-OSL (Electron Spin Resonance - Optically Stimulated Luminescence), OSL, U/Th (Uranium-Thorium) dating techniques and palaeontological determinations was homogenic, about 0.065 m ka^−1^ during the last 1 Ma.

*Tectonic background*. The new derangement (Stage 5) indicated the start of the new phase of the fluvial system evolution managed first of all by regional uplift called the ‘recovery phase’ [115] or ‘continental scale uplift’ [103]. This followed the cessation of subduction and termination of the foreland cycle. In the present study, it can be termed ‘cratonic uplift’ continued hitherto. It deserves special study but already available data allow highlighting of its main features.

Sokolovskii & Volkov [116] identified a substantial positive inversion of the crustal movements encompassing the southeastern part of the East European platform beginning from the end of the Middle Sarmatian Substage. By their estimation, the total maximal amplitude of the movements since the end of the Oligocene Epoch was over 350 m in the area of interest, which is one of the greatest within the platform. However, in addition, it differed from the regional style of the cratonic vertical movements, the pattern of which was already established during the previous foreland period.

The erosion rates noted above obtained for the Dniester, Meuse, Seine, Yonne and Somme rivers confirmed the view of Gibbard & Lewin [117] about accelerated incision over about 400 ka during the Middle Pleistocene in Europe. This also corresponds to Molnar’s [118] concept concerning the increase in accumulation rates of terrestrial sediment globally in the late Cenozoic. The incision rates are considered as the most reliable proxy of uplift rates, especially for long intervals, e.g. Maddy [119]. Whether this uplift is partly attributable to the long-scale regional true-tectonic trend or isostatic rebound of the crust attributed to the mid-Pleistocene climatic degradation (e.g. [1]), or their superimposition [15] is a subject for debate.

There is a prominent general dip of the modern surface and all Late Miocene formations in a south–south-southeast direction that is portrayed in sections [44, figs 3, 4]. Most tributaries of the northern part of the Podillia Upland follow this dip (figure 17e). The crest of the Badenian–Lower Sarmatian barrier reef (figure 1c) decreases in the same direction. All this evidence indicates an obvious regional tectonic tilt.

A superficial view on the areal topography shows that it differs from the internal regions of the platform by increased ruggedness and segmentation on regions of different height, slope, slopes’ aspect and orientation of the erosional landforms. It was conditioned by tectonic partitioning. This largely determines morphological differences between the valley’s Reaches 1, 2, 3 and their segments. One of the probable explanations of the coincidence of the main knickpoints of the modern Dniester River profile with the reaches boundaries (§5.4) is the different rate of the vertical crustal movements of tectonic regions, preserved up to the present.

The modern fluvial pattern of Reach 1 is an inherited frozen cast of its oldest counterpart. The common entrenchment was 20−50 m deep there, which is 5 to 10 times less than in Reaches 2 and 3. The lack of the upper alluvial members in Reach 1 suggests that the incision of the valley also had naked character. The distinct asymmetry of the valley within Reaches 2 and 3, as well as its right-sided shift, can be explained by its passing through the boundaries between the noted tectonic regions. In the area of the Balta Formation, the general surface dip is broken by regional heightening and so that maximal heights coincide with maximal thicknesses of the formation and vice-versa [44] figures 3, 4, 17 and 18. This attests to the isostatic component of the crustal movements.

*Rock erodibility*. The Dniester valley is a prominent example of an obvious influence of the marked difference in rocks’ erodibility on the morphology of the valley (Reaches 2, 3 and the upstream part of Reach 4). The appearance of the incised meanders and canyon in comparison to the gentle upper macro-slope is tightly associated with entrenchment of the river in harder rocks. More local is the effect of the river crossing the barrier reefs composed of the hard limestones, narrowing of the valley and intensification of meandering.

There are so-called weathering crusts over different carbonate rocks, from the compacted carbonate gruss to massive clays. These rocks as deeply weathered deposits of the Red-Brown Formation and loesses are the most erodible. Being involved in fluvial erosion their finest materials increase the suspended and dissolved load. When fluvial erosion reached the stratum of the Tyras Formation (Badenian age), composed of gypsum and anhydrides, this provided impetus to the development of karst (continuous hitherto) also increasing the dissolved load.

*Climatic background*. Unlike the previous period, the stratigraphy which includes several hiati, the cratonic period can be called continuous. This continuity implies a constant river runoff against the background of a more or less constant slope. Judging from the increase of alluvial deposits coarseness the effective discharge of this runoff also slowly increased through the Pliocene–Pleistocene to the Holocene.

This observation contradicts the contraction of the Dniester basin (very close to the modern one, figure 17d) in the post-Baltinian period and the relatively stable position of the Carpathians, which experienced isostatic uplift and erosion [120] and which were the undisputed main concentrator and supplier of orographic precipitation. There are also no data to indicate any increase in regional precipitation in the plain part of the basin, located in the extra-glacial zone. Probably the reason should be sought in the change in the flow regime, namely in its contrast, with an increase in effective discharges during floods as a result of more abundant and intense rainfall episodes and/or snowmelt. The direct short-term effects of the continental Pleistocene glaciations on the fluvial development have been discussed in §6.1.3 and can be characterized in terms of so-called ‘upstream control’ [121]. As was noted before concerning formation of alluvial funs [41], physical weathering and gravitational processes accompanying development of mountain glaciation and permafrost during cold periods intensified mobilization and delivery of sediment.

*Secondary processes*. Serious impact on the valley morphology was made owing to the hillslope linear and sheet erosion and accumulation of deposits of the Red-Brown Formation, loesses and their slopewash derivatives on the valley sides. Samodurov [42] showed the wide distribution of the latter as overlying more ancient alluvial deposits all along the valley. These colluvial and aeolian deposits modified valley slopes smoothing their initially sharp erosional stepwise features in many places. It is assumed that the loess supply occurred by means of global dust transportation/fallout as well as by deflation of the alluvial deposits from the valley floor and their deposition within the valley as well as adjacent to it [27].

The Dniester valley experienced both modes but the second is unexpected for the canyon bottom, where loess even overlies alluvial deposits of the lowest terraces, in several places. The mechanism of this process has remained unclear so far. Most intensification of the aeolian processes is associated with the end of the Middle and Late Pleistocene cold intervals under the conditions of a bare surface and/or sparse vegetation [27]. That was also the time of increasing linear erosion and mass wasting on the valley sides.

#### 6.3.3. Valley development within transition zone ‘platform–Black Sea depression’

*Fluvial development*. Reach 4 of the Dniester valley as a transitional zone between sediment-transfer river and receiving basin has a number of specific (with reference to upstream counterparts) features. Some (the notable increase of the valley slope flattening, gradual general decreasing of entrenchment, break or pinching out of the alluvial macro-units, interchange of alluvial and estuarine sedimentary environments, presence of overdeepenings and burial alluvial plain) are obviously related to the proximity of the basin and most often accounted for by fluctuations of its level arising from eustatic changes. However, except drainage of the Black Sea shelf during the maximum of the last glaciation, other traces of the sea-level fall and rise cannot be reliably tied to this explanation. The connections of the Black Sea with the world ocean in the Pliocene-Quaternary is an unresolved issue ([122] with references herein).

*Base-level oscillations*. Among the hypotheses regarding the Messinian draw-down of the Black Sea, the dramatic climatic change that led to a sharp reduction of rivers’ input is considered as one of the main factors [92]. During the present study, the evidence of such deep aridization is not found. Moreover, the hypothetical correlation of the incision related to the Dniester Liman Series with the Messinian fall in the Black Sea level (§6.1.3) suggests a significant discharge of water from the Dniester at this time.

The Roksolany Formation recorded a decline in sea level in the Gelasian Stage, affecting the coastal part of the valley and compensated by alluvial filling and aggradation, interspersed with estuarine invasions. During this time, the alluvial and estuarine units formed an extensive coastal plain occupying a significant proximal part of the modern shelf. This plain was eroded (at least twice) in the Middle Pleistocene (Palaeo-Euxinian high-level stage of the Black Sea) far inland, more than 100 km from the modern coastline. Previously, estuarine deposits have been recorded within the adjacent Prut valley at the same latitude as the Tiraspol locality [96]. The last near-mouth overdeepening and its partial infilling took place during sea-level fall in the Late Pleistocene (§6.1.3). The latest oscillations of the sea-level in the Holocene Epoch created the modern estuaries of the northwestern Black Sea coast including the Dniester Liman.

*Tectonic background*. Until recently, the area of this reach was thought as a monocline at the margin of the East European platform [123], i.e. reflecting the same tectonic tilt which is characteristic of the area of the Dniester basin in general (§6.3.2). However, evidence from available seismic data, indicates that there was increasing subsidence towards the outer Black Sea shelf in the Pliocene Epoch followed by aggradation and advance of the shelf in the Quaternary Period [92–94,122,124–126].

As previously summarized [13], the subsidence within the coast involved the folding of the Pontian and older rocks in the Pliocene as well as vertical block movements along Late Miocene faults that continued into the Pliocene–Pleistocene epochs. Thereby, a vast coast-shelf area adjoining the slope of the Black Sea depression bears traces of active neotectonics. It is likely that it is a hinge-flexure zone at the contact between the platform and depression with oscillatory small-amplitude differentiated movements along shifting hinge lines. This is an alternative solution of the numerous ingressions/regressions as well as a complicated plan view of the lowest Dniester valley reach with a widening-narrowing pattern.

## 7. Conclusion

This article presents the results of the multidisciplinary study of the medium-sized, continental-scaled, bedrock degrading valley of the Dniester River throughout its plain course. This river system comprises a markedly long and complex history, which although specific to this system, provides a sequence of fluvial evolution characteristic of the southern-western part of the East European Plain. The valley system is distinguished by rich and accessible geological materials in the form of outcrops and descriptions of borehole cores in alluvial deposits as well as by one of the most detailed studies in comparison with other such type valleys of the world. Nevertheless, the thorough analysis of scientific heritage revealed significant methodological and conceptual shortcomings in previous approaches, the latter based on outdated paradigms with limited methodology. These touched upon the shallow study of the alluvial deposits proper, fluvial processes, stratigraphy and as consequence, the somewhat simplistic ideas concerning the valley’s development. This new analysis has demonstrated the strength of the detailed sedimentological reconstruction that the stepped morphologies are not identical to the river’s terrace staircase and their pure morphological identification with a fixed quantity throughout the valley that ‘numerical’ correlation may lead to incorrect interpretations. To overcome these flaws, the authors based their concept on a comprehensive sequential study of alluvial lithofacies, their associations and architectural elements. This allowed differentiation of numerous fragmentary alluvial members (elementary allostratigraphic units) in much greater and varying quantities than previously. It is established that these members are preserved remnants of a continuous stepped alluvial blanket on the valley flanks. In some cases, the distinguishable difference between steps is never more than 1.5 m. The members themselves have their specific lithofacies features and different dimensions.

Developing the above concept, the members were combined into macro-units according to their affiliation to the definite valley reach (segment), altitudinal range, set of facies and their association as well as the source provenance of the alluvial clasts and grains. Each macro-unit was interpreted in terms of the fluvial style. This concept was integrated with detailed morphological analysis of the valley, both as a whole and each of its reaches with application of different topographical sources including the newest ones. For the first time, to our knowledge, the boundaries of the valley were outlined, its main morphometric parameters were established, and the largest of the terraces were identified.

The evidence obtained led to revision of a number of earlier reconstructions and to presentation of a new evolutionary scheme based upon stratigraphy, sedimentology, geomorphology, tectonics and palaeogeography. This scheme, as part of a broader hypothesis of the development of the fluvial system of the Eastern Carpathian foreland [23,41,44,54], essentially supplemented it. The refined integrated stratigraphy with clear priority of the criteria preserved in its previous framework, was elaborated by using the combined principles of allostratigraphy, litho-facies division and correlation as well as adding the potential correlation with regional and continental-scale events. As such this stratigraphy replaces the previous ‘detailed’ scheme of 6−14 terrace stages (only for the Pliocene-Quaternary period and elaborated on material only from the third and fourth reaches) with a much more objective scheme consisting of two periods (late Miocene and Pliocene–Pleistocene) and seven stages, including all reaches of the Dniester valley.

The growing Eastern Carpathian proto-land in the Para-Tethys Sea gave rise to the Dniester and other conjugate fluvial systems, in common with other major rivers in the region, including the Don, the Dnieper and the Volga. During most stages of the late Cenozoic, it was the main water- and sediment-source area for them. The ancient platform land played a subordinate role except during stages 4 and 6 when the area of river basins increased from its side. The fluvial system passed through the stages of accelerated ‘naked’ erosion, distributive and tributive process activity. Some were accompanied by degradational and aggradational episodes. The fluvial system history included relatively long periods of stability and shorter spans of reorganization, triggered, in common with the rivers, by external forcing.

The morphology of the Dniester valley is characterized by its significant depth (up to 250 m), for the deepest of the valleys of the East European Plain; relative narrowness; irregular longitudinal profile with numerous inflections; non-uniform asymmetric cross-profile, prominent canyon and incised meanders in the deepest part of the central reaches. It also includes a number of lithological members’ fragments, most of which are buried beneath a blanket of hillslope and aeolian deposits. All this has its own features in framing each of the valley’s four reaches.

Regarding the sedimentation aspect, the Dniester and its system for most of its history were associated with transfer of sediment and intermittent accumulation. This was essentially characteristic during the last cratonic period. The alluvial members scattered over the valley’s flanks are remnants of this general tendency. However, the Conglomeratic and Balta formations are contrasting examples of fluvial development, with a profound aggradation trend and storing considerable part of the inland sediment flux. This also accounts for the buried coastal alluvial plain and locally for near-mouth river overdeepenings in the coastal region.

The variety of clastic alluvial lithofacies reflects no less diversity of the transportation and depositional modes: water-current, hyperconcentrated and debris flows. Comparing them and their associations with their known late Cenozoic analogues of the valleys of the inner regions of the East European Plain [12,13] all gravel lithofacies and their successions, including the main sand-dominated successions, are unique or encountered very rarely to the east of the Dniester.

The Dniester and other confluent streams in its basin repeatedly change their fluvial morpho-sedimentary style through their history and along the valley in response to external conditions. There were sand- and gravel-dominated streams, free- and constrained meandering, braided as well as downfilling and backfilling examples. Judging by their sedimentary thicknesses and facies differences the effective channel-forming discharge and solid load varied significantly. In the authors' concept of the development of the fluvial system, tectonics is the leading external underlying control in conditions of changeable but generally favourable climatic conditions influencing fluvial processes. This control was provided by the regime of tectonic movements: uplift—quiescence in the Carpathians; subsidence—flexural deformations in the foreland during the foreland period. This, in turn, changed gradient, increased the height of the Carpathian land, shifted and tilted the depositional zones in the foreland forcing the fluvial system to adapt. Throughout this period the Dniester drainage basin area remained lowland. In addition, during the formation of the Balta Delta its area was subjected to weak subsidence accompanied by an average infilling depositional rate of –0.025 to 0.95 m ka^−1^.

The cratonic period was marked by generally accelerated uplift and tilt towards the Black Sea, including a eustatic contribution and tectonic partitioning. This caused an increase of the reaches’ specificity and transformation of most of the lowland to upland. The incision rate also increased during this period from 0.045 m ka^−1^ up to 0.144 m ka^−1^ (late Chibanian–Holocene) in the valley’s middle reaches.

The Pliocene-Quaternary sedimentary record near the Black Sea coast is evidence of numerous oscillations of the base-level of erosion, the amplitude of which reached as much as 500 m. These resulted in fluvial system progradation and retrogradation within the approximately 300 km strip of the Black Sea depression coastal-shelf-slope zones. Among the causes, there is evidence of general subsidence of the depression, probable small-scale hinge-flexural deformations at the margin of the platform and unidentified climatic changes.

The climatic control principally reflects precipitation change influencing stream discharge and competence. Currently, there is no reliable historical data to clarify this issue. However, it can be assumed that mountains were the environmental determinants in this point, their growth and expansion increasingly attracting atmospheric moisture, and exporting it through the drainage systems. At the same time, the shift of the climatic zones, the superimposed cold/warm periodicity in the Quaternary were secondary factors, the role of which in the development of river valleys in the extra-glacial zone remains unclear.

The global climatic changes, especially during the Quaternary gave rise to such phenomena as glaciation, permafrost and processes of loess formation. The first of these are associated with short but strong effects of intense glaciofluvial erosion and sediment supply during Middle Pleistocene continental glaciation. This penetrated to the most upstream plain reach of the valley. An increase of river discharge and sediment flux was probably favoured owing to development of permafrost in the Carpathians [127] and mountain glaciation during the Younger Dryas Stadial there [26]. The most significant result of loess formation was smoothing of the valleys’ slopes and essential transformation of the primary fluvial relief reached maximum during the Late Pleistocene Subepoch [27,128]

As for the impact of rock erodibility, two forms of influence have been observed. One is related to the relative weakness of flysch-molasses rocks of the Carpathian-derived sedimentation as the main supplier of detrital material to the Dniester system. The second form is the differing resistance of the valley’s bedrock to fluvial erosion, which caused local changes in the transverse and longitudinal profiles of the valley, the form of incised meanders, among others.

The model of the Dniester valley presented is far from being complete, but, as presented here, it creates a quite new construction for its further study and discussion being an impressive example of long-term major river development (over at least 11 Ma) under the foreland-cratonic conditions. New data on this valley are in themselves the key to understanding the specifics of a wide variety of geological processes occurring at the contact of active tectonic belts and platforms. It was this position that ensured its singularity differed from the valleys of the inner platform regions (of the rivers Volga, Don, Dnieper [12]) but specific to other valleys within the same tectonic region (Prut and Siret [41,54]).

## Data Availability

The part of the datasets supporting this article are from the archive (STATE RESEARCH AND DEVELOPMENT ENTERPRISE 'STATE GEOLOGICAL INFORMATION FUND OF UKRAINE', SRDE 'GEOINFORM UKRAINE', Kyiv, Ukraine). Mapping data are from digital images of SRTM https://www.usgs.gov/centers/eros/science/usgs-eros-archive-digital-elevation-shuttle-radar-topography-mission-srtm (data recieved 2023.10.11) [29]. Supplementary material is available online [129].
